# Peptide-based covalent inhibitor of tubulin detyrosination promotes mesenchymal-to-epithelial transition in lung cancer cells

**DOI:** 10.1073/pnas.2514990123

**Published:** 2025-12-31

**Authors:** Hathaichanok Impheng, Ghislain Gillard, Nuttanid Numnoi, Anthony Feral, Matthieu Simon, Maxime Louet, Muriel Amblard, François Juge, Lubomir Vezenkov, Krzysztof Rogowski

**Affiliations:** ^a^Department of Physiology, Faculty of Medical Science, Naresuan University, Phitsanulok 65000, Thailand; ^b^Tubulin Code Team, IGH (Institute of Human Genetics) UMR 9002, Genetics, Cell Biology and Development department, Univ Montpellier, CNRS, Montpellier 34090, France; ^c^IBMM (Institut des Biomolécules Max Mousseron), Amino Acids, Peptides and Proteins department, Université de Montpellier, CNRS, Ecole Nationale Supérieure de Chimie de Montpellier, Montpellier 34293, France

**Keywords:** microtubules, detyrosination, vasohibins, inhibitors, cancer

## Abstract

Detyrosination is a reversible posttranslational modification specific to α-tubulin, which has been implicated in cancer progression and invasiveness by promoting epithelial-to-mesenchymal transition. The members of the vasohibin family, VASH1 and VASH2, were previously identified as the first class of enzymes involved in catalyzing this modification. Here, we report the development of a covalent VASH inhibitor, which is characterized by high specificity and low toxicity. By combining the use of a new compound with molecular approaches in lung cancer cell lines, we find that tubulin detyrosination plays an important role in the maintenance of mesenchymal properties. We show that in the absence of VASH activity, collective cell migration and 3D spheroid formation are severely compromised. Moreover, we demonstrate that the observed phenotypes are caused by the accumulation of the important epithelial marker E-cadherin with simultaneous reduction in mesenchymal markers N-cadherin and vimentin. Taken together, our study establishes tubulin detyrosination as a promising target for the future development of anticancer treatment.

## Significance

The vast majority of cancers are derived from epithelial origin. The key mechanism which contributes to cancer progression and leads to metastasis is called epithelial-to-mesenchymal transition. It involves alteration in expression of important markers including downregulation of E-cadherin and upregulation of vimentin and N-cadherin. These changes result in more efficient cell migration and colony formation, a prerequisite for metastatic invasion. Here, using lung cancer cell lines as a model, we show that inhibition of a tubulin posttranslational modification called detyrosination reverts epithelial-to-mesenchymal transition. Importantly, reduced detyrosination increases the levels of E-cadherin with concomitant reduction of vimentin and N-cadherin, which strongly affects cell migration and colony formation. Taken together, our data establish detyrosination as a promising target for anticancer therapy.

Microtubules (MTs) are essential cytoskeletal elements composed of α/β-tubulin heterodimers that are involved in various important functions including cell division, morphogenesis, intracellular transport, and cell motility. One of the central mechanisms allowing for the functional adaptations of MTs includes a number of unusual posttranslational modifications. The majority of tubulin modifications targets the C-terminal tails, which are present on the MT surface where they provide essential interaction sites for MT-associated proteins (MAPs) and molecular motors. The most prevalent among them is detyrosination, a reversible modification which is specific to α-tubulin and consists in the removal of the very C-terminal tyrosine leading to the generation of so-called Δ1-tubulin. The forward reaction is catalyzed by members of the vasohibin family (VASH1 and VASH2) complexed with the small vasohibin binding protein (SVBP) (1, 2) and Tubulin MetalloCarboxyPeptidase 1 (TMCP1) (3) also known as MATCAP (4). In contrast, the reverse reaction is accomplished by the Tubulin Tyrosine Ligase (TTL) (5). As such, the level of detyrosination in any given cell is established as a result of competition between the forward and reverse enzymes (6).

Detyrosination has been shown to play an important role in various processes such as mitosis (7), cell migration (8), neuronal development (9, 10), cardiac mechanotransduction, and myogenesis (11), and its deregulation was found to be associated with numerous diseases including neurodegeneration (12), cardiomyopathies (13), heart failure (14), and cancer (15, 16).

At the molecular level, detyrosination acts as a positive signal for the binding and activity of various motor proteins including kinesin-1 (9, 10) and kinesin-7/CENP-E (7). In contrast it serves as a negative regulator for the binding of CAP-Gly domain containing proteins such as Cytoplasmic Linker Protein (CLIP-170, CLIP-115) and p150^glued^, a component of the dynein/dynactin complex (17). Furthermore, it also inhibits the activity of MT-depolymerizing kinesin-13 motors (18, 19) and the binding of Echinoderm-Microtubule associated protein Like 2 (EML2) (20). Despite the numerous readers of detyrosination that have been already identified, a recent study revealed the existence of additional readers that remain to be uncovered (20).

Previous studies have shown that detyrosination accumulates during epithelial-to-mesenchymal transition (EMT) and might promote this process although its exact role remains unclear (16, 21). EMT is a highly coordinated cellular program essential for embryogenesis and tissue development (22). However, it is also involved in metastasis and cancer progression by promoting cell migration and invasiveness leading to the establishment of secondary tumors (23). EMT is characterized by changes in cell morphology caused by decreased expression of epithelial markers such as E-cadherin (E-cad) and concomitant accumulation of mesenchymal markers such as N-cadherin (N-cad) and vimentin (24). The reduction in E-cad expression weakens cell–cell interactions resulting in a more elongated cell morphology, which facilitates cell motility and metastasis while the presence of vimentin filaments protects the cells from mechanical stress (25).

Accumulating evidence suggests that VASHs play an important role in promoting EMT in various cancers including breast (26, 27), ovarian (28), pancreatic (29), cervical (30), and hepatocellular carcinoma (31). Thus, to directly address the role of these enzymes in EMT, we have developed a potent and highly specific VASH inhibitor named LV80. Treatment of A549 lung cancer cells with this new compound increased E-cad and decreased N-cad and vimentin levels, affecting cell migration and spheroid formation. These phenotypes were recapitulated in cells knockout for both VASHs (VASH1+2KO). Remarkably, knockout of TTL in VASH1+2KO cells restores detyrosination level and rescues these phenotypes. We further show that depletion of E-cad partially rescues the cellular phenotypes of VASH1+2KO cells. Our results show that tubulin detyrosination plays an important role in the maintenance of the mesenchymal state by negatively regulating E-cad levels.

## Results

### Development of LV80, a Potent and Specific VASH Inhibitor.

**Fig. 1.**
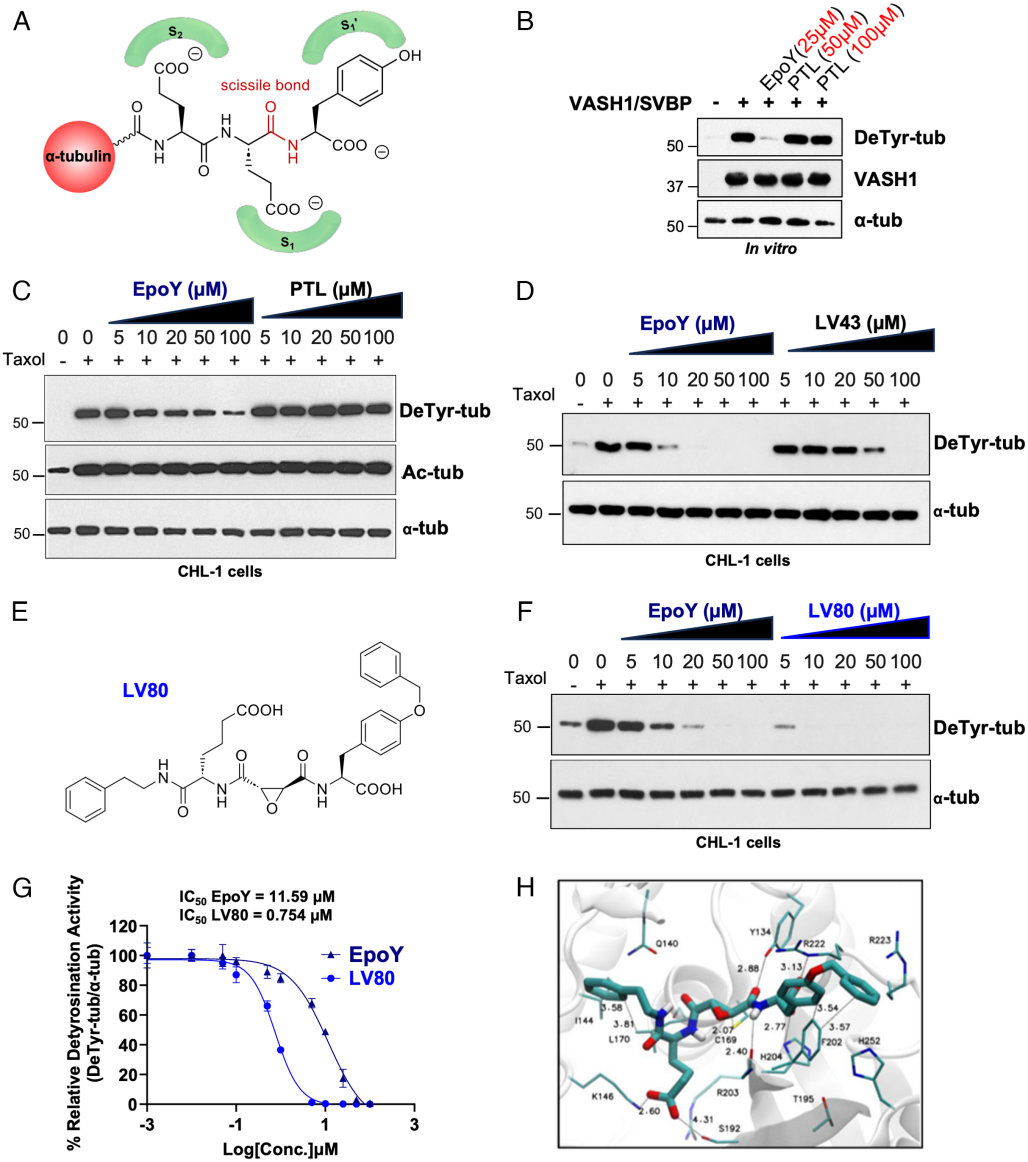
Development of LV80, a potent and specific VASH inhibitor. (*A*) Drawing of α-tubulin C-terminal tail showing substrate recognition and cleavage site. S1′ depicts the C-terminal tyrosine while S1 and S2 refer to two glutamate residues. Scissile bond refers to the covalent bond that can be cleaved by the detyrosinase. (*B*) EpoY but not parthenolide (PTL) triggers a decrease in VASH1/SVBP-dependent detyrosination activity in vitro as shown by immunoblot analysis. The concentrations of EpoY and PTL are indicated in red. (*C*) Increasing concentrations of EpoY but not PTL trigger a decrease in Taxol-induced detyrosination in CHL-1 cells as shown by immunoblot analysis. Note that EpoY and PTL treatments do not affect the levels of tubulin acetylation. (*D*) Immunoblot analysis of Taxol-induced detyrosination levels in CHL-1 cells treated with LV43. Increasing concentrations of LV43 inhibit detyrosination less efficiently than EpoY. (*E*) Chemical structure of LV80. (*F*) Immunoblot analysis of the effect of LV80 treatment on Taxol-induced detyrosination levels in CHL-1 cells. Increasing concentrations of LV80 decrease detyrosination levels more efficiently than EpoY treatment. (*G*) Concentration–response curve for EpoY and LV80. (*H*) Molecular docking of LV80 into VASH1 active site revealing the putative binding mode (hydrogen bonds).

The identification of VASHs as tubulin detyrosinases involved the use of a covalent epoxide-based inhibitor inspired by the C-terminal sequence of α-tubulin (Fig. 1*A*) (2). Previously, we synthesized a series of compounds in which the highly reactive epoxide residue has been fused to increasing numbers of amino acids corresponding to α-tubulin C-terminus (-Y, -EY, or -EEY). Among them only EpoY covalently inhibited VASH activity in vitro and reduced detyrosination levels in cellulo (*SI Appendix,* Fig. S1*A*) (2). Initially, we compared the inhibitory efficiency of EpoY with a widely used inhibitor of detyrosination called parthenolide (PTL) (32). Interestingly, the structure of PTL also contains an epoxide residue, which suggests potentially overlapping inhibitory mechanisms (*SI Appendix,* Fig. S1*B*). Using our previously established in vitro assay involving fully tyrosinated MTs from Sf9 cells (33), we found that while EpoY efficiently inhibited VASH activity, PTL showed no effect even at a high concentration (Fig. 1*B*). To further extend our analysis, we tested the two inhibitors in cellulo, using human CHL-1 cells in which detyrosination is catalyzed primarily by VASHs (1). In this assay the inhibitors are coincubated with Taxol to induce high levels of detyrosination, which is most likely caused by MT lattice expansion (34) as shown by concomitant accumulation of tubulin acetylation (Fig. 1*C*). As such, the majority of detected detyrosination is generated de novo by VASHs on expanded MTs. In agreement with the in vitro data, EpoY reduced tubulin detyrosination in a dose-dependent manner without affecting tubulin acetylation while PTL had no effect even at high concentrations (Fig. 1*C*). Taken together, our results clearly demonstrate that PTL does not inhibit VASHs and its effect on tubulin detyrosination is most likely mediated through MT destabilization resulting from direct binding to tubulin as previously demonstrated (35).

Considering that EpoY suffers from several drawbacks including low affinity and potential binding to other targets (2), we optimized it using medicinal chemistry. Based on iterative chemical variations coupled to standardized in vitro assay, we developed a series of compounds (36) including one we have termed LV43. To generate this compound, we maintained the tyrosine residue and the epoxide function at the P1′ and P1 positions respectively, while the ethyl ester group present at the P2 position of EpoY was substituted with a homologated glutamate (*SI Appendix,* Fig. S1*A*). In the in vitro assay, LV43 showed a more potent inhibition of VASH1 activity as compared to EpoY (*SI Appendix,* Fig. S1*C*). However, in a cell-based assay, LV43 was less efficient than EpoY (Fig. 1*D*). The difference between the two assays suggested reduced cell penetration of LV43. Thus, to further increase the affinity and to improve cell penetration of LV43, we generated additional compounds by adding hydrophobic aromatic groups, which led to the development of LV80 (Fig. 1*E* and *SI Appendix,* Fig. S1*A*). In the in vitro assay, we found that LV80 inhibited VASH1 and VASH2 activity more efficiently than EpoY (*SI Appendix,* Fig. S1 *D-E*). Strikingly, LV80 was also more effective in the cell-based assay (Fig. 1*F*) with an IC_50_ of 11,59 µM for EpoY compared to 0,75 µM for LV80 (Fig. 1*G* and *SI Appendix,* Fig. S1*F*). These data show that introduction of aromatic groups increased not only the affinity of the inhibitor but also likely its cell penetration.

To explain the superior efficacy of LV80 as compared to EpoY and LV43, we employed molecular docking to predict their lowest energy binding poses. Our analysis was aided by a recent study which resolved the structure of VASH1 cocrystalized with EpoY (37). We docked the inhibitors in their noncovalent forms to assess their affinity for VASH1 before the irreversible covalent reaction with the active site cysteine took place (*SI Appendix,* Fig. S1 *G* and *H*). Lower-scoring poses revealed that in contrast to EpoY, LV43 and LV80 engaged K146 and S192 of VASH1 via their acidic tails (Fig. 1*H*). In addition, LV80 also interacted with I144 and L170 through its phenyl group and engaged in strong hydrophobic π–π staking interactions with F202 via its benzyl moiety. As such, the specific interactions of LV80 provide a potential mechanistic explanation for the greatly enhanced properties of this inhibitor.

### Characterization of LV80 Inhibitory Properties.

**Fig. 2.**
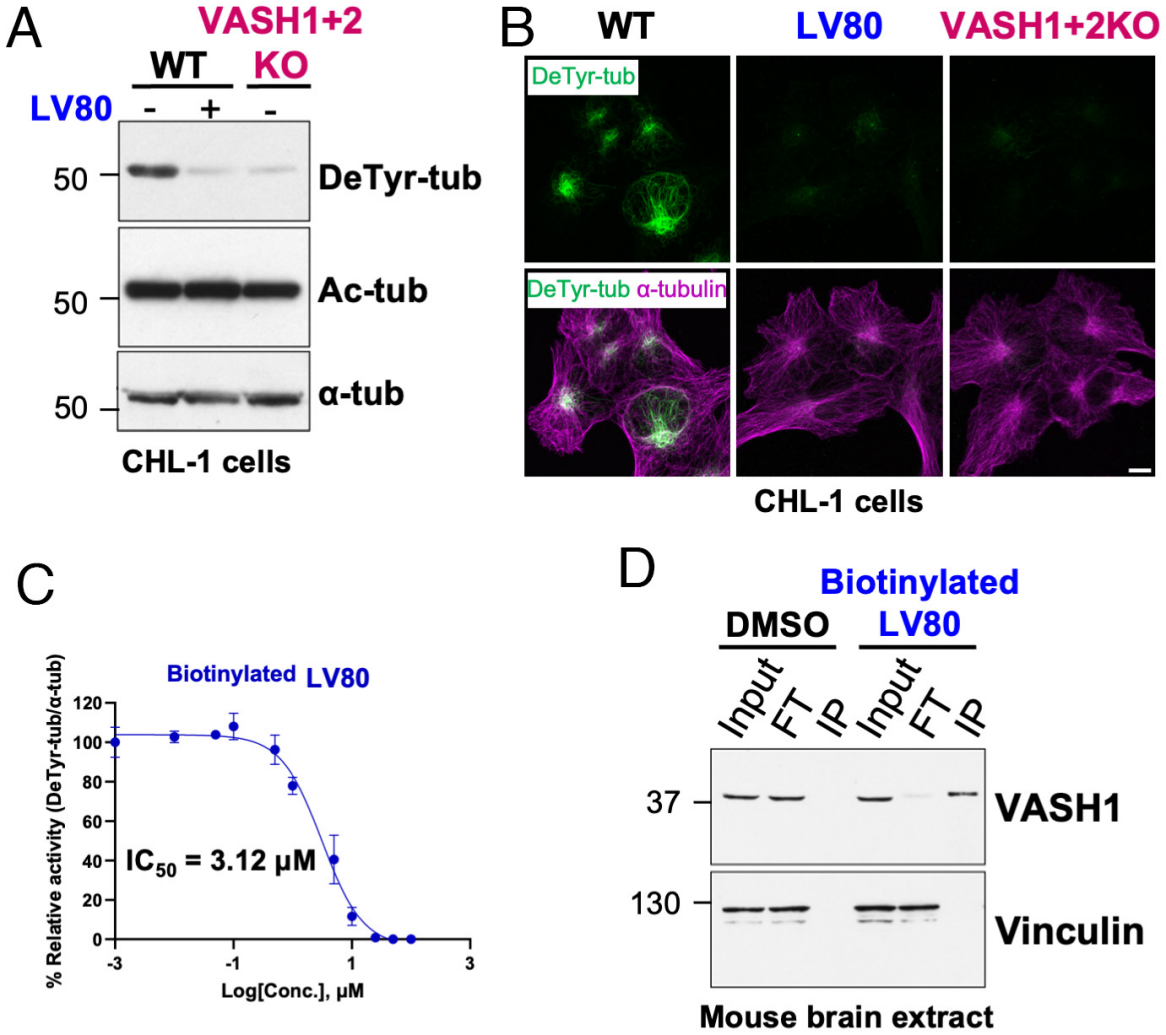
Characterization of LV80 inhibitory properties. (*A*) Immunoblots of protein extracts from CHL-1 cells. Treatment with LV80 lowers detyrosination levels down to the levels of detyrosination observed in CHL-1 cells knockout for VASH1 and VASH2 (VASH1+2KO). Note that LV80 treatment does not affect the levels of acetylated tubulin. (*B*) Immunofluorescence analysis of CHL-1 cells incubated with LV80 shows a strong reduction in endogenous detyrosination levels similar to the one observed in VASH1+2KO cells. (Scale bar, 10 µm.) (*C*) Concentration–response curve for biotinylated LV80. (*D*) Pull-down assay of endogenous VASH1 from mouse brain protein lysates using biotinylated LV80 reveals target engagement between LV80 and VASH1.

Following the identification of the LV80 compound, we tested its ability to inhibit endogenous detyrosination. We treated wild type (WT) CHL-1 cells with LV80 and compared the level of detyrosinated tubulin to its level in VASH1+2KO cells (1). Immunoblot and immunofluorescence analyses showed that the treatment of WT cells with LV80 reduced detyrosination to a similar level found in VASH1+2KO cells (Fig. 2 *A*-*B*). These data demonstrate that LV80 completely inhibits VASH activity in cellulo.

Next, we tested whether the inhibitory effect of LV80 is mediated by its direct binding to VASH1 and VASH2, its presumed targets. Thus, we modified the inhibitor by the covalent attachment of biotin (*SI Appendix,* Fig. S1*A*). Using our standard in cellulo assay, we found that biotinylated LV80 inhibits VASH activity with only slightly reduced potency (Fig. 2*C* and *SI Appendix,* Fig. S2*A*). To show direct target engagement of the inhibitor, we performed GFP-dependent pulldowns of tagged VASH1 and VASH2 from HEK293 cells treated with biotinylated LV80. Using streptavidin conjugated Horseradish Peroxidase (HRP), we detected VASH1 and VASH2 in the samples treated with biotinylated LV80 but not in the untreated controls demonstrating direct covalent binding of the modified inhibitor to VASHs (*SI Appendix,* Fig. S2*B*). Subsequently, we used biotinylated LV80 on protein extracts derived either from mouse brain or HEK293 cells. We found that modified LV80 efficiently precipitated VASH1 from both types of extracts (Fig. 2*D* and *SI Appendix,* Fig. S2*C*) establishing it as an efficient tool for future analysis of VASH’s binding partners.

Next, we compared the enzymatic selectivity of EpoY and LV80 toward other cysteine proteases. We chose two biologically relevant representatives, calpain and caspase-3. We observed that both compounds showed a slight inhibition of calpain activity exclusively at the highest concentration while they had no effect on the caspase activity regardless of the concentration used (*SI Appendix,* Fig. S2 *D* and *E*). These data support the selectivity of EpoY and LV80 toward VASHs.

Next, since the structure of LV80 was inspired by the sequence of the α-tubulin C-terminal tail we tested whether it might inhibit other tubulin tail-processing enzymes such as CCP1, CCP5, and TMCP1. We also included CarboxyPeptidase A (CPA) in our analysis because before the discovery of VASHs, it was frequently used to generate detyrosinated MTs for in vitro assays (10, 37). We observed that LV80 had no effect on the activity of any of these enzymes (*SI Appendix,* Fig. S2 *F–I*), providing further evidence for its enzymatic selectivity.

Finally, we assessed the viability of CHL-1 cells in the presence of increasing doses of LV80, PTL, and Taxol. We found that in contrast to PTL and Taxol, which showed dose-dependent toxicity, the treatment with LV80 had no effect on cell viability even at the highest concentration (*SI Appendix,* Fig. S2*J*). Taken together, these properties of LV80 make it the most powerful and specific inhibitor available to date to determine the function of VASH-dependent detyrosination in cellulo.

### Role of Tubulin Detyrosination in the Maintenance of the Mesenchymal State.

Previous studies have shown that EMT is accompanied by the accumulation of tubulin detyrosination (16, 21), suggesting a role in this process. Furthermore, it remains unclear whether detyrosination is also required for the maintenance of the mesenchymal state. Having developed a specific VASH inhibitor, we assessed its ability to reverse the mesenchymal characteristics using the A549 lung cancer cell line as a model.

Initially, we compared the toxicity of the LV80 inhibitor either on A549 or on a noncancerous IMR-90 lung-derived cell line. In agreement with our results in CHL-1 cells, the treatment with increasing doses of LV80 had no effect on the cell viability of either cell line, even at the highest concentration tested (*SI Appendix,* Fig. S3 *A* and *B*). These results further confirmed that our inhibitor exhibits low toxicity.

**Fig. 3.**
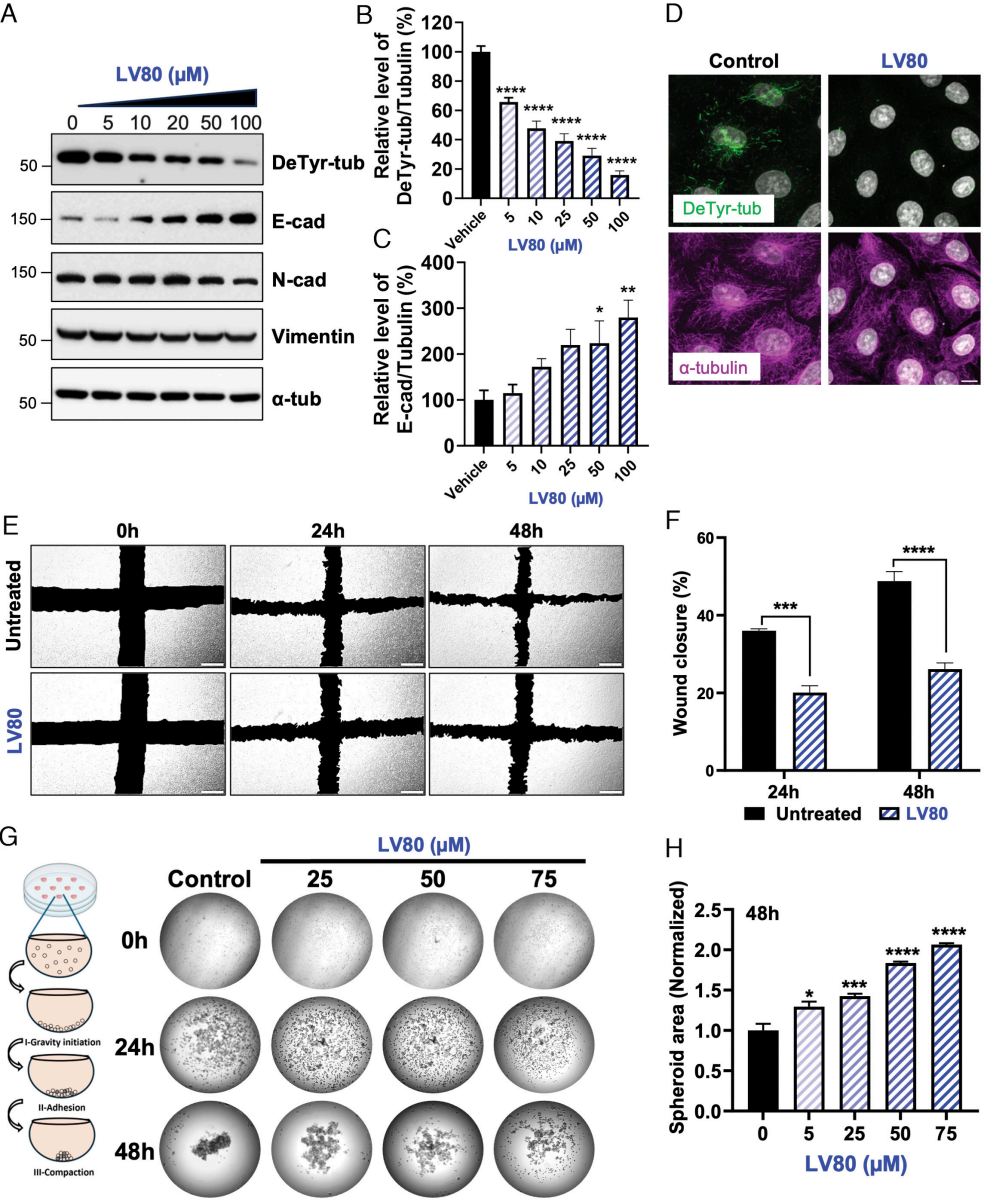
Role of tubulin detyrosination in the maintenance of the mesenchymal state. (*A*) Immunoblots of protein extracts from A549 cells treated with increasing concentrations of LV80. Note that incubation with LV80 lowers the levels of Taxol-induced detyrosination but also triggers an increase in the levels of E-cad. (*B*) Quantification of detyrosination levels in A549 cells treated with increasing concentrations of LV80. (*C*) Quantification of E-cad protein levels in A549 cells treated with increasing concentrations of LV80. (*D*) LV80 treatment inhibits detyrosination in A549 cells as shown by immunofluorescence analysis. (Scale bar, 10 µm.) (*E*) Treatment of A549 cells with LV80 affects cell migration as revealed by wound healing assay. (*F*) Percentage of wound closure in WT or LV80-treated A549 cells. (*G*) Schematic drawing depicting stages of spheroid formation in the hanging drop assay (*Left*). Increasing concentrations of LV80 prevent spheroid formation (*Right*). (*H*) Normalized spheroid area after 48 h of treatment with LV80.

Subsequently, we treated A549 cells with LV80 and evaluated its effect on detyrosination as well as on the expression of several important markers associated with the EMT process. We observed that the addition of the inhibitor strongly reduced the level of detyrosinated tubulin in a dose-dependent manner as shown by immunoblot and immunofluorescence analyses (Fig. 3 *A*, *B*, and *D*). Strikingly, we observed a strong accumulation of E-cad (Fig. 3 *A* and *C*) accompanied by a modest reduction in the levels of N-cad and vimentin (Fig. 3*A* and *SI Appendix,* Fig. S3 *C* and *D*). The effects of LV80 on detyrosination and E-cad were further confirmed in three additional lung cancer-derived cell lines including H1299, H520, and H1975 (*SI Appendix,* Fig. S3 *E–J*). These data suggest a universal involvement of detyrosination in the regulation of EMT, at least in lung cancer cells.

Next, considering the changes in the levels of cadherins, we tested the speed of cell migration in the presence or absence of LV80 using a wound healing assay. We found that the addition of the inhibitor strongly reduced the ability of A549 cells to close the wound (Fig. 3 *E* and *F*). Similar results were obtained in H1299 cells (*SI Appendix,* Fig. S3 *K* and *L*), demonstrating that tubulin detyrosination is required for efficient cell migration.

Finally, we assessed the ability of the cells to form 3D spheroids in a hanging drop assay. While untreated A549 cells efficiently formed compact spheroids, the addition of LV80 inhibited spheroid formation in a dose-dependent manner (Fig. 3 *G* and *H*). These observations were further validated in H1299 cells (*SI Appendix,* Fig. S3 *M* and *N*). Since we observed the accumulation of detyrosinated tubulin in suspended A549 cells over time (*SI Appendix,* Fig. S3 *O* and *P*), it provides a potential explanation for the strong effect of the inhibitor on spheroid compaction. Taken together, our results show that detyrosination plays an important role in 3D spheroid formation.

### Generation and Phenotypical Characterization of A549 VASH Knockout Cell Lines.

**Fig. 4.**
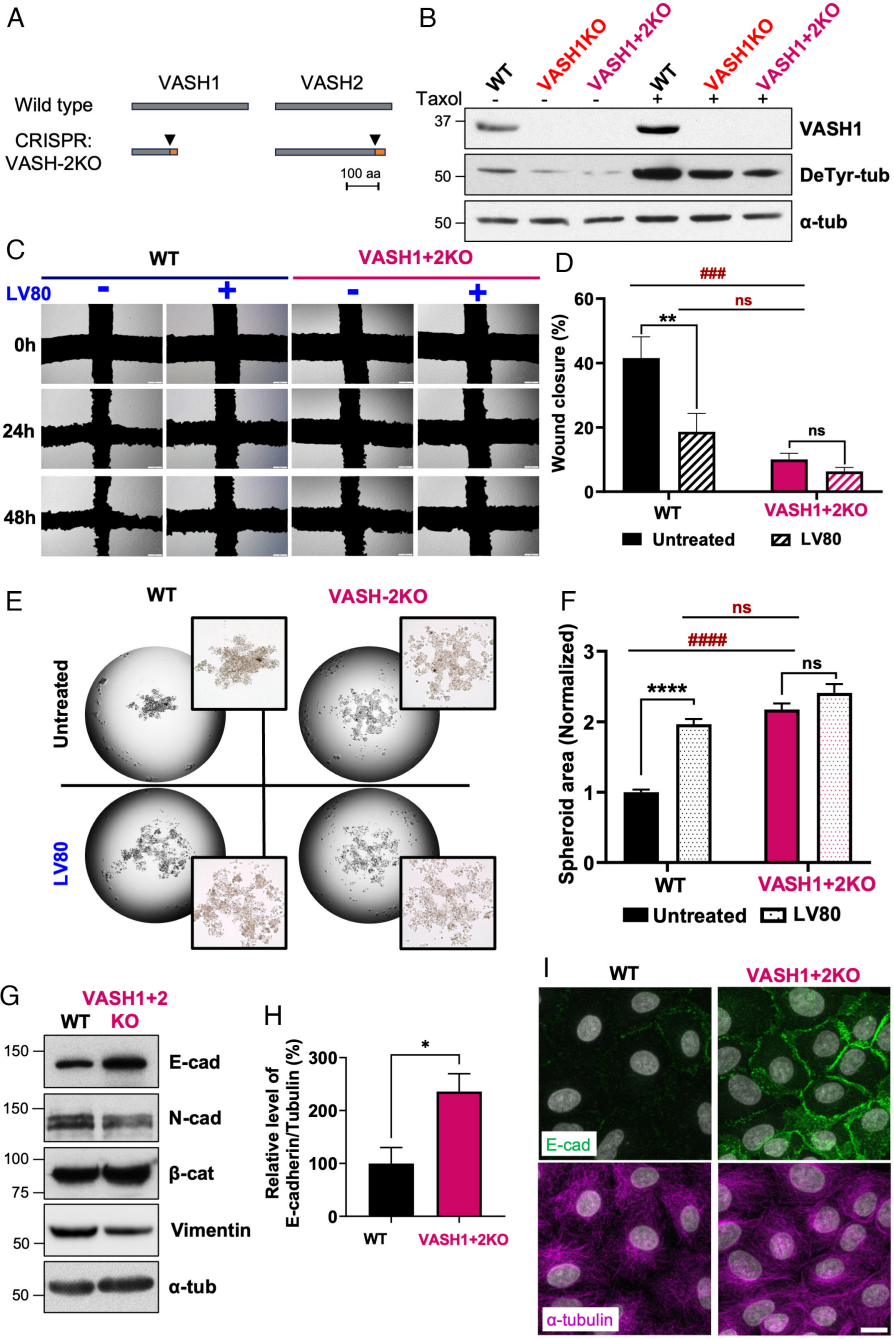
Generation and phenotypical characterization of A549 VASH knockout cell lines. (*A*) Schematic drawing depicting either the full length or truncated versions of VASH1 and VASH2 generated by CRISPR/Cas9 in A549 cells. (*B*) Immunoblot analysis of protein extracts from WT, VASH1-KO, and VASH1+2KO or the same cell lines treated with Taxol. (*C*) Comparison of the migration speed between WT and VASH1+2KO cells in the presence or absence of LV80 using wound healing assay. (*D*) Percentage of wound closure in WT or VASH1+2KO cells treated or not with LV80 for 48 h. Note that the addition of LV80 in VASH1+2KO cells does not affect their migration. (*E*) Comparison of the ability of WT or VASH1+2KO cells to form a compact spheroid in the presence or absence of LV80 after 48 h using hanging drop assay. (*F*) Normalized spheroid area in WT or VASH1+2KO cells treated or not with LV80. (*G*) Immunoblot analysis of protein extracts from WT or VASH1+2KO cells. Note the increase in E-cad levels. (*H*) Quantification of E-cad protein levels (% E-cad/Tubulin) in WT or VASH1+2KO cells. *(I*) Immunofluorescence analysis of WT or VASH1+2KO cells colabeled for E-cad and α-tubulin. Note the accumulation of E-cad in the VASH1+2KO cells. (Scale bar, 15 µm.)

To confirm our observations with the LV80 inhibitor, we generated knockout cell lines lacking either VASH1 (VASH1-KO) or VASH1 and VASH2 (VASH1+2KO) using CRISPR/Cas9 technology. The sequencing of the mutated loci revealed either deletions or insertions, with all of them resulting in the introduction of premature stop codons (Fig. 4*A* and *SI Appendix,* Fig. S4 *A* and *B*). In agreement, the VASH1+2KO cells showed reduced expression of both VASHs at the mRNA level, most likely due to nonsense-mediated mRNA decay (*SI Appendix,* Fig. S4*C*). In addition, we also confirmed the lack of VASH1 expression in the single and double knockout cell lines at the protein level with a specific antibody (Fig. 4*B*). As a consequence, the analysis of detyrosination revealed a strong reduction in this modification already in VASH1-KO cells that was further exacerbated in the VASH1+2KO line (Fig. 4*B* and *SI Appendix,* Fig. S4*D*). The difference between the WT and knockout cell lines became even more apparent in the presence of Taxol (Fig. 4*B* and *SI Appendix,* Fig. S4*D*). Thus, our data show that VASHs are the major detyrosinases expressed in A549 cells although not the only ones as evidenced by the presence of remaining detyrosination in VASH1+2KO cells that is most likely catalyzed by TMCP1. To confirm this hypothesis, we treated the WT and knockout cell lines with LV80 in the presence of Taxol. This led to a marked reduction of detyrosination in WT and VASH1-KO but not VASH1+2KO cells (*SI Appendix,* Fig. S4 *E* and *F*). Since LV80 does not inhibit TMCP1 (*SI Appendix,* Fig. S2*F*), our results are consistent with this enzyme being responsible for the generation of residual detyrosination. Furthermore, they confirm complete elimination of the VASH-dependent detyrosinase activity in VASH1+2KO cells.

Having developed VASH1+2KO cells, we next analyzed their migration speed in a wound healing assay. We found that in comparison to WT, VASH1+2KO cells migrated significantly slower with a speed that was comparable to WT cells in the presence of LV80 (Fig. 4 *C* and *D*). As expected, the addition of the inhibitor to VASH1+2KO cells had no effect on their migration speed (Fig. 4 *C* and *D*). These data demonstrate that the effect of LV80 on cell migration is mediated through VASH inhibition.

Next, we assessed the ability of the VASH1+2KO cells to form a compact spheroid in the hanging drop assay. While the WT cells showed efficient compaction after 48 h, the VASH1+2KO cells, similarly to the WT cells treated with LV80, remained dispersed (Fig. 4 *E* and *F*). Again, the addition of the inhibitor to VASH1+ 2KO cells had no effect on the compaction (Fig. 4 *E* and *F*). Thus, the effect of LV80 on spheroid formation stems from its ability to inhibit VASHs.

Since the treatment with the VASH inhibitor led to an increase in E-cad and a decrease in N-cad as well as vimentin levels, we assessed the expression of these EMT markers in VASH1+2KO cells. In agreement with the inhibitor data, we found that the knockout cells had elevated levels of E-cad, while the amount of N-cad and vimentin were reduced (Fig. 4 *G*–*I* and *SI Appendix,* Fig. S4 *G* and *H*).

In accordance with elevated E-cad levels, we also observed the accumulation of β-catenin (Fig. 4*G*), which is the cytosolic partner of E-cad that upon activation translocates to the nucleus and drives the expression of genes associated with cell proliferation such as cyclin D1 and C-myc. First, we showed that the increased levels of β-catenin do not lead to nuclear accumulation, arguing against an activation of the β-catenin signaling pathway (*SI Appendix,* Fig. S4 *I* and *J*). In addition, since the activation of β-catenin is mediated through its dephosphorylation, we labeled the cells with antibodies specific to dephosphorylated β-catenin. We found no difference in the level of dephosphorylated β-catenin between the WT and VASH1+2KO cells (*SI Appendix,* Fig. S4 *K* and *L*). Accordingly, the levels of downstream effectors of activated β-catenin including cyclin D1 and C-myc remained mostly unaffected (*SI Appendix,* Fig. S4 *M–P*). Taken together, our data show that although the accumulation of E-cad leads to an increase in the amounts of β-catenin, it does not trigger its activation.

### Rescue of the VASH1+2KO Phenotypes by TTL Knockout in A549 Cells.

Since detyrosination is reversible, we argued that knocking out TTL in the VASH1+2KO cells should increase the levels of detyrosination due to the presence of unopposed TMCP1 activity and as such rescue the phenotypes caused by the absence of VASHs. Initially, we generated and characterized TTL knockouts in WT A549 cells. The complete loss of TTL was confirmed by immunoblot analysis in two independent clones resulting from the introduction of premature stop codons (*SI Appendix,* Fig. S5 *A* and *B*). The knockout of TTL led to almost complete loss of tyrosinated tubulin with a concomitant accumulation of detyrosination (*SI Appendix,* Fig. S5 *B* and *C*). Unexpectedly, we observed a strong increase in E-cad at the protein level (*SI Appendix,* Fig. S5*D*), which could be partially reduced by the treatment with LV80 (*SI Appendix,* Fig. S5 *E–G*). These results suggest that abnormally high levels of detyrosination, similarly to its depletion, promote E-cad accumulation.

**Fig. 5.**
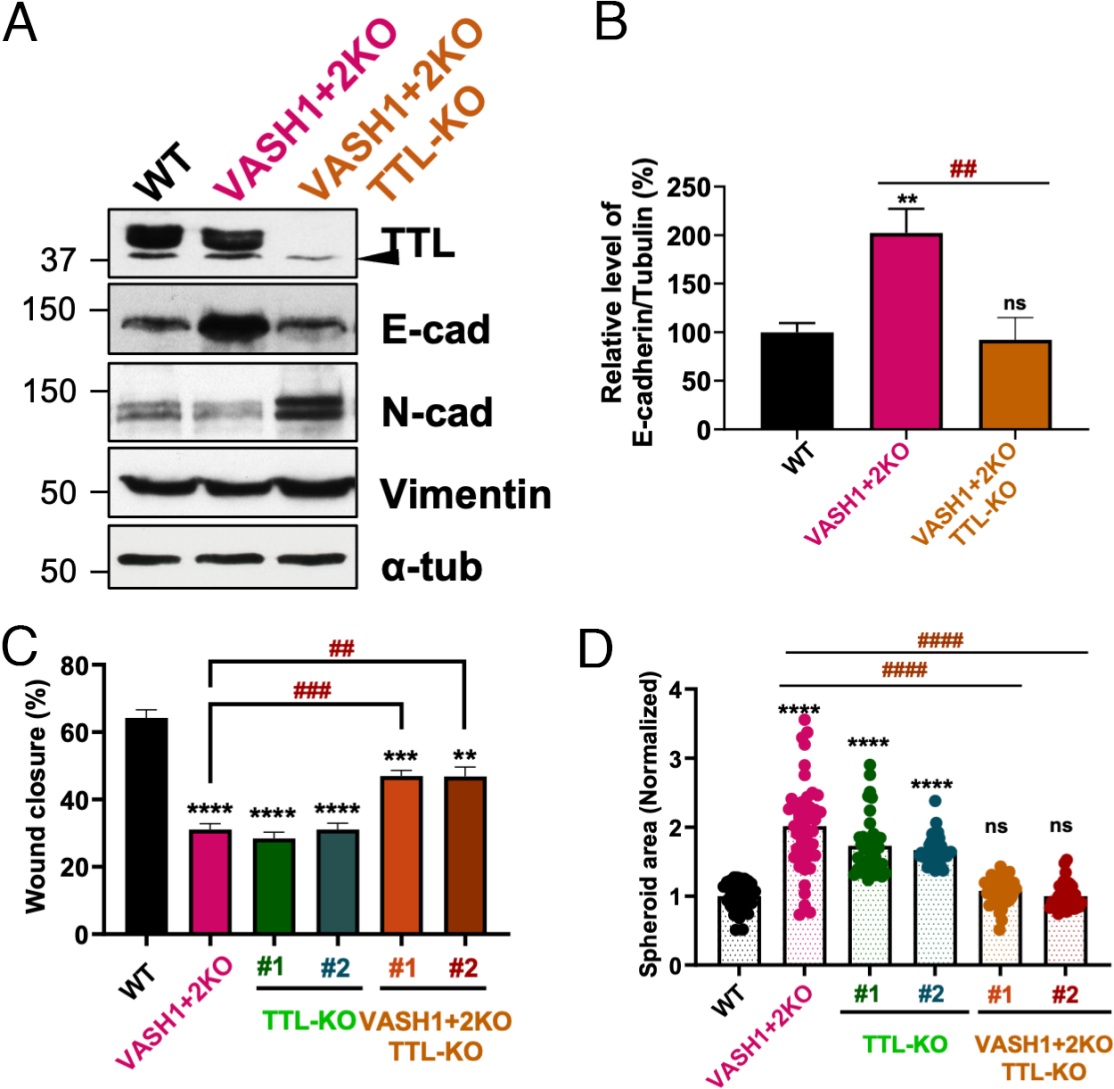
Rescue of the VASH1+2KO phenotypes by TTL knockout in A549 cells. (*A*) Immunoblot analysis of protein extracts from WT, VASH1+2KO, and VASH1+2KO/TTL-KO A549 cells. The arrowhead points at an unspecific band recognized by the anti-TTL antibodies. (*B*) Quantification of E-cad protein levels (% E-cad/Tubulin) in WT, VASH1+2KO, and VASH1+2KO/TTL-KO cells. (*C*) Percentage of wound closure in WT, VASH1+2KO, TTL-KO, and VASH1+2KO/TTL-KO cells. (*D*) Normalized spheroid area in WT, VASH1+2KO, TTL-KO, and VASH1+2KO/TTL-KO cells.

Next, we knockout TTL in VASH1+2KO cells obtaining two independent clones, which as expected lacked TTL and showed increased amounts of detyrosination (*SI Appendix,* Fig. S5 *H–J*). Surprisingly, the level of tyrosinated tubulin was not affected (*SI Appendix,* Fig. S5 *H–J*), suggesting only a modest increase in detyrosination. In addition, we found that VASH1+2KO cells lacking TTL showed reduced levels of E-cad, which were comparable to WT cells (Fig. 5 *A* and *B*). Next, we assessed the wound healing ability of the single and double knockout cells. We observed that TTL-KO cells exhibited reduced migration speed that was comparable to VASH1+2KO cells, while the double knockout cells migrated significantly faster (Fig. 5*C* and *SI Appendix,* Fig. S5*K*). Accordingly, the reduced spheroid compaction displayed by VASH1+2KO and TTL KO cell lines was also rescued in cells concurrently devoid of both types of enzymes (Fig. 5*D* and *SI Appendix,* Fig. S5*L*). Taken together, we show that restoring detyrosination in VASH1+2KO cells through the removal of TTL leads to almost complete rescue of all the phenotypes caused by the lack of this modification.

### Reduced Tubulin Detyrosination Inhibits Lysosome-Dependent E-cad Degradation.

**Fig. 6.**
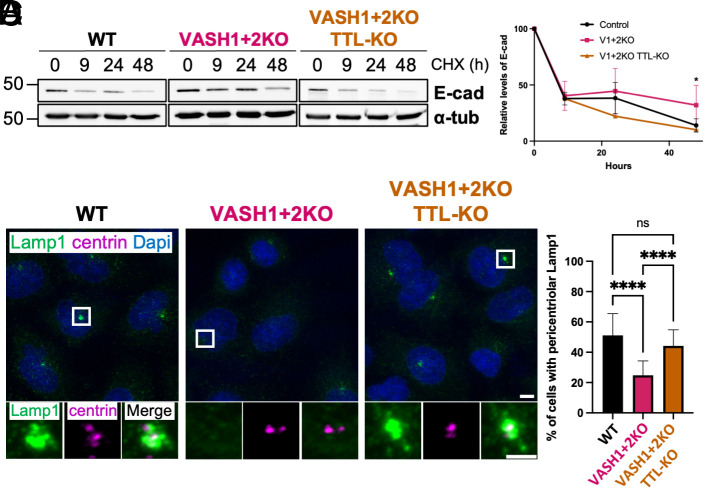
Reduced tubulin detyrosination inhibits lysosome-dependent E-cad degradation. (*A*) Immunoblot analysis of protein extracts from WT, VASH1+2KO, or VASH1+2KO/TTL-KO A549 cells treated with cycloheximide (CHX) for the indicated times. (*B*) Quantification of E-cad protein levels in A549 cells upon treatment with CHX. (*C*) Immunofluorescence analysis of WT, VASH1+2KO, or VASH1+2KO/TTL-KO cells colabeled for Lamp1 and centrin. Note the loss of pericentriolar accumulation of Lamp1 in VASH1+2KO cells. (*D*) Percentage of cells exhibiting Lamp1 pericentriolar accumulation.

Next, we investigated the mechanism responsible for the accumulation of E-cad in VASH1+2KO cells. One well-established pathway by which cancer cells regulate E-cad expression is through transcriptional repression by core EMT transcription factors, including SNAIL, TWIST1, ZEB1, and ZEB2 (38). However, since we did not observe an increase in the expression of E-cad at the mRNA level (*SI Appendix,* Fig. S6 *A* and *B*), the accumulation of E-cad is likely due to a stabilization at the protein level. Considering that a steady-state protein abundance is determined by the balance between synthesis (translation) and degradation, we assessed E-cad turnover using cycloheximide chase assay. This analysis revealed that E-cad is degraded less efficiently in VASH1+2KO cells as compared to WT or the rescue VASH1+2KO/TTL-KO cells (Fig. 6 *A* and *B*), suggesting that tubulin detyrosination controls the amounts of E-cad by modulating its degradation.

A recent study has found that MT detyrosination plays a role in the degradation pathway by regulating lysosome-autophagosome fusion (39), prompting us to examine the organization of these organelles. To investigate potential defects in the autophagic flux, we analyzed the levels of p62 and LC3-II, two markers known to accumulate as a result of defective autophagy. Our immunoblot analysis showed no difference in the amount of these two proteins (*SI Appendix,* Fig. S6*C*), suggesting that the autophagic flux is not affected. These observations were further confirmed using BafilomycinA1 to block autophagy. This treatment led to similar accumulation of p62 and LC3-II in WT, VASH1+2KO, and rescue cells (*SI Appendix,* Fig. S6*D*) further indicating that detyrosination does not play an important role in the regulation of autophagy in A549 cells.

Next, we analyzed the organization of lysosomes using Lamp1 as a marker. While in WT and rescue cells Lamp1-positive lysosomes showed mainly a pericentriolar accumulation, in VASH1+2KO they exhibited a diffused localization (Fig. 6 *C* and *D*). Similar lysosomal organization was observed in the WT cells treated with LV80 (*SI Appendix,* Fig. S6 *E* and *F*). Taken together, our data suggest that defective lysosomal localization is likely to be responsible for the E-cad accumulation in both VASH1+2KO and LV80-treated cells.

### Evaluation of the Role of E-Cadherin in Cell Migration and Spheroid formation.

**Fig. 7.**
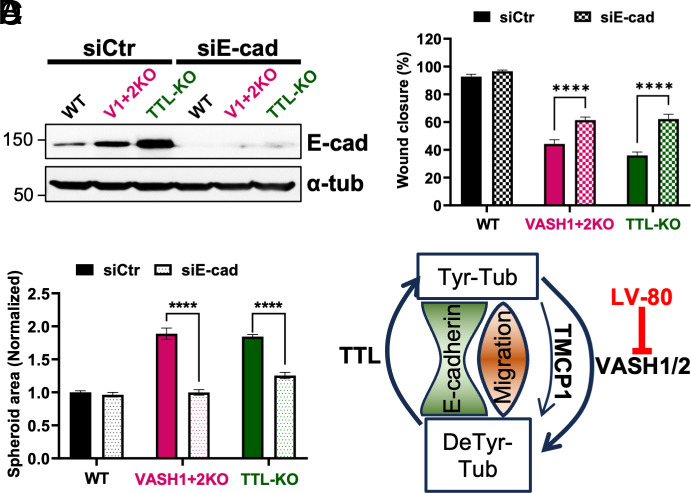
Evaluation of the role of E-cadherin in cell migration and spheroid formation. (*A*) Immunoblot analysis of protein extracts from WT, VASH1+2KO, or TTL-KO A549 cells transfected with either control (siCtr) or E-cad (siE-cad) siRNA. (*B*) Percentage of wound closure in WT, VASH1+2KO, and TTL-KO A549 cells. (*C*) Normalized spheroid area in WT, VASH1+2KO, and TTL-KO cells transfected either with siCtr or siE-Cad. (*D*) Graphical summary of the interplay between detyrosination and E-cad levels correlated with cell migration

Our results show that the migration speed of the VASH1+2KO and TTL-KO cells is strongly reduced in comparison to control cells. Strikingly, both knockouts are characterized by high levels of E-cad, which is particularly prominent in cells at the migrating edge (*SI Appendix,* Fig. S7*A*). Thus, we hypothesized that the elevated levels of E-cad might be at least partially responsible for slower migration. To test this idea, we performed siRNA-dependent knockdown of E-cad and assessed its effect on cell migration in the WT and the two knockout cell lines. The knockdown efficiently reduced the expression of E-cad in all cell lines as demonstrated by immunoblot and immunofluorescence analysis (Fig. 7*A* and *SI Appendix,* Fig. S7 *B* and *C*). While the knockdown of E-cad had no effect on the migration of WT cells, it significantly improved the wound healing ability of VASH1+2KO and TTL-KO cell lines (Fig. 7*B* and *SI Appendix,* Fig. S7*D*). These results show that increased expression of E-cad is at least partially responsible for the reduced migration speed of the VASH1+2KO and TTL-KO cells.

Next, considering the well-established role of E-cad in cell–cell interactions, we tested whether it also plays a role in spheroid formation. We found that knockdown of E-cad efficiently rescued spheroid formation in both VASH1+2KO and TTL-KO cell lines (Fig. 7*C* and *SI Appendix,* Fig. S7*E*) suggesting that one of the most important functions of detyrosination is the regulation of E-cad levels. The interplay between detyrosination and E-cad levels correlated with their effect on cell migration are graphically summarized in Fig. 7*D*.

## Discussion

During recent years tubulin detyrosination has emerged as a relevant therapeutic target for various diseases including cancer (15, 40). However, there are currently very few reliable inhibitors of the enzymes catalyzing this modification. Here, we describe the development and characterization of a highly potent and specific inhibitor of VASHs, the first class of detyrosinating enzymes to be identified (1, 2). The discovery of these enzymes has been made possible using irreversible peptide-like inhibitor called EpoY (2). Initially, we compared the inhibitory properties of EpoY and parthenolide, which was originally discovered in a screen of a library of natural extracts and subsequently widely used as a detyrosination inhibitor (7, 32, 41, 42). Since the identification of parthenolide predates the discovery of VASHs, the inhibitory activity in cells was not directly validated in vitro (1, 2, 32). The presence of epoxide residue in both EpoY and parthenolide, suggests that the two molecules might share a similar inhibitory mechanism. However, our data demonstrate that, unlike EpoY, parthenolide does not inhibit VASH activity in vitro nor in cellulo. These findings are supported by a recent study indicating that the effect of parthenolide on detyrosination is likely indirect since it binds to tubulin and destabilizes MTs (35). This raises the question of why, despite both inhibitors containing epoxide residue, only parthenolide appears to bind to tubulin. One possible explanation is the presence of α, β-unsaturated lactone in the structure of parthenolide, a second highly reactive group required for the inhibition of detyrosination (32). Thus, it is likely that the effect of parthenolide on MT stability is mediated through the formation of covalent bonds between this group and cysteine or histidine residues present in both tubulins. Taken together, we do not recommend the use of parthenolide as an inhibitor of detyrosination. However, a recent study has shown that at lower concentrations, PTL can be used as an efficient inhibitor of mitosis by affecting kinetochore attachment to MTs (43).

Starting with EpoY as a lead molecule, we performed its optimization using medicinal chemistry aided by molecular docking. We exploited the chemical space around the epoxide core of EpoY to improve its activity, selectivity, and physicochemical properties with a particular focus on cell penetration. We also modified the tyrosine residue by the addition of a second benzene ring to improve the affinity of the inhibitor toward VASHs and to prevent the potential binding of the inhibitor to tyrosine kinases as previously reported for EpoY (2). These experiments led to the development of LV80, which is more efficient at inhibiting VASHs than EpoY not only in vitro but also in cellulo. The newly developed inhibitor is characterized by low IC_50_, falling in the nanomolar range, and no detectable toxicity when tested on various cell lines or even highly sensitive primary neuronal cultures (patent WO2023025861). In addition, LV80 has also recently been found to show promising effects in vivo (44). These features of LV80 are in stark contrast to parthenolide, which in accordance with its MT-depolymerizing activity was found to be highly toxic (35). Furthermore, the LV80 inhibitor displayed specificity toward VASHs over other cysteine proteases or other types of proteases involved in the processing of the tubulin C-terminal tails such as TMCP1, the second class of detyrosinases (3, 4). Moreover, using a biotinylated version of the inhibitor we confirmed that the binding of LV80 to VASHs is covalent as was the case for EpoY (2). In the future, modified LV80 can serve as an easy-to-use tool to identify interactors of VASHs from any given cell line or tissue. This important area of research remains greatly unexplored with SVBP being the only known binding partner of these medically relevant enzymes (1, 2, 45).

Previous works have shown that detyrosinated tubulin accumulates during EMT and might promote this process (16, 21). We used our highly specific VASH inhibitor to determine the role of detyrosination in the maintenance of the mesenchymal state using the A549 lung cancer cell line as a model. The choice of the cell line was dictated by its epithelial origin and the expression of mesenchymal markers such as N-cad and vimentin (46). Moreover, tubulin-targeting drugs such as Taxol are commonly used in lung cancer therapy providing validation for MTs as an effective target for the development of future treatments (47). Since the newly developed inhibitor targets the enzymes responsible for tubulin modifications instead of MTs themselves, it represents an alternative approach with potentially lower toxicity to modulate MT activity. Furthermore, as recently demonstrated for EpoY (15), it could even be used in combination to potentiate the effects of Taxol. The proposed synergy may seem counterintuitive since a previously published model proposed that promoting MT detyrosination through TTL inhibition has an additive effect with Taxol by enhancing MT stability, therefore making them resistant to kinesin-13-mediated depolymerization (48). Although at this point, the mechanism synergizing Taxol treatment with VASH inhibition is not clear, our data show that hyper- or hypodetyrosination can lead to the same outcome as demonstrated for E-cad protein levels.

To assess the function of detyrosination at the cellular level, we employed wound healing assay to measure collective 2D migration. We found that reduced detyrosination, regardless if it is through pharmacological inhibition or genetic ablation of the VASHs, strongly affected the wound healing ability of the cells. Surprisingly, similar defects were also observed in TTL-KO cells, which are characterized by abnormally high levels of detyrosination. These data are consistent with a recent study which, using one-dimensional micropattern assay to evaluate the migration of RPE-1 cells depleted either for VASHs or TTL shows that cells with either reduced or abnormally high levels of detyrosination fail to undergo directed cell migration (8). These defects were attributed to a loss of detyrosination-dependent cell polarization. The authors propose that symmetry breaking required for cell polarization is orchestrated by a positive feedback loop driven by kinesin-1-dependent transport taking place specifically on detyrosinated MTs. In most cells, the majority of detyrosinated MTs are found on one side of the nucleus that corresponds to the localization of the centrosome (49). This asymmetry is consistent with previously reported pericentrosomal localization of VASHs (50), which together with their preferences for polymers results in a localized formation of modified MTs (1, 2). In the absence of detyrosinated MTs, as is the case in cells lacking VASH activity, kinesin-1-dependent transport is no longer biased toward the migrating edge and cells fail to polarize and to exert directional movement. On the other hand, in the absence of TTL, which has a strong preference for tubulin dimers (51), detyrosinated tubulin derived from depolymerization of modified MTs is no longer retyrosinated. This leads to a random incorporation of detyrosinated tubulin into all MTs and an eventual loss of symmetry breaking. As such, the key element of their model is the asymmetrical distribution of detyrosinated MTs. However, we observed that in the VASH1+2KO devoid of TTL, despite all MTs carrying detyrosination, the migration was partially rescued. This suggests that although the asymmetrical distribution of detyrosinated MTs plays an important role in individual cell migration in confined environments, it is not the only mechanism involved in this phenomenon during collective cell migration. Accordingly, we found that reducing the abnormally high levels of E-cad also ameliorated the wound healing ability of VASH1+2KO or TTL-KO cells pointing to the negative role of this protein in cell migration.

As a complementary approach, we used hanging-drop assay to evaluate the ability of the cells to form compact 3D cellular spheroids, which is the first step required for malignant invasion (52). We found that the cells were highly sensitive to VASH inhibition and failed to form well-organized spheroids even in the presence of a low concentration of the inhibitor. These results were further confirmed in the VASH1+2KO cells. One possible explanation for a particular sensitivity of the cells in the drop assay is a substantial increase in the level of detyrosination that we observed in cultures grown in suspension. These results are consistent with previously published reports showing that tubulin detyrosination increased up to 30 times in mammary epithelial cells grown without attachment (53). Detyrosinated tubulin appears to be enriched in cellular protrusions called microtentacles, which play an important role in cell–cell and cell–matrix attachments (16, 54). These protrusions are particularly abundant on circulating tumor cells, underscoring the potential relevance of detyrosination in metastasis (55). Importantly, defective spheroid formation observed in VASH1+2KO cells was completely rescued by the knockout of TTL while partial rescue was also obtained by the knockdown of E-cad. Taken together these results show that tubulin detyrosination plays an important role in spheroid formation and one of its most important functions is the regulation of E-cad levels as was the case in wound healing assay. Since the expression of E-cad mRNA in inhibitor-treated cells as well as VASH1+2KO cells was not increased, it suggests a stabilization at the protein level. Accordingly, we observed a reduced degradation rate of E-cad in VASH1+2KO.

A recent study has shown that detyrosinated MTs serve as platforms required for spatial concentration of lysosomes and for their downstream fusion with autophagosomes that is necessary for the initiation of degradation (39). Therefore, one way by which reduced detyrosination could lead to an increase in E-cad levels is by decreasing the frequency of fusion between lysosomes and autophagosomes. However, we found that the autophagic flux was not affected in VASH1+2KO cells suggesting a normal rate of vesicular fusion at least for lysosomes and autophagosomes. In contrast, we observed a strong reduction in pericentriolar localization of Lamp-1-positive lysosomes in both inhibitor-treated and VASH1+2KO cells, which is consistent with the enrichment of lysosomes on detyrosinated MTs previously described in BS-C-1 cells (39) and the implication of detyrosination in lysosome motility in neurons (56). Lysosomes travel on MTs in a stop-and-go pattern due to the opposing activities of kinesin and dynein motors (57). As such, the role of detyrosination in the pericentriolar accumulation of lysosomes could stem from its importance in the regulation of their transport toward the minus-end of MTs or their anchoring in the vicinity of the centrioles. Interestingly, the luminal pH of lysosomes depends on their position within the cells with peripheral lysosomes exhibiting a more alkaline pH associated with a decreased activity of lysosomal cathepsin (58). Thus, the reduced amount of pericentriolar lysosomes observed in LV80-treated and VASH1+2KO cells with diminished detyrosination, could specifically affect endo-lysosomal degradation and explain increased E-cad levels. Future experiments should be aimed at testing this model.

In conclusion, we describe the development of a potent and highly specific inhibitor of VASH-dependent detyrosination. Through the combined use of the newly developed compound and genetic approaches, we demonstrate that tubulin detyrosination plays an important role in maintaining the mesenchymal characteristics in lung cancer cell lines. We show that inhibiting or depleting VASH activity strongly affects cell migration and spheroid formation, underscoring the potential importance of detyrosination in metastasis. Furthermore, we show that the effect of detyrosination is mediated through changes in the levels of E-cad, the highly relevant marker associated with EMT. As such, our work establishes VASHs as a promising target for anticancer therapy and opens up the possibility to directly address the role of detyrosination in various cancers. Finally, the excellent physicochemical and biological characteristics of the newly developed inhibitor make it suitable for future use in animal studies.

## Materials and Methods

### Cell Culture, Generation of Knockout Cell Lines and siRNA.

All cell lines and culture conditions as well as knockdown and knockout strategies including their validation are detailed in *SI Appendix*.

### Incubation with Drugs and Inhibitors.

The incubation times and concentrations used for each drug or inhibitor used in the present study are detailed in *SI Appendix*.

### Immunofluorescence Analysis.

Immunofluorescence labeling is detailed in *SI Appendix*, including the list of antibodies as well as the protocol for the quantification of fluorescence intensities.

### Immunoblot Analysis.

After SDS-PAGE, proteins from cell extracts were transferred onto nitrocellulose membranes, then incubated with primary antibodies in blocking solution. The detailed protocol and antibodies are described in *SI Appendix*.

### Droplet Digital PCR.

Expression levels of E-cad and VASHs were quantified using ddPCR as described in *SI Appendix*.

### Biochemistry.

Information relative to pull-down experiments, in vitro assays as well as expression and purification of recombinant proteins are described in *SI Appendix*.

### Wound Healing and Hanging Drop Assays.

Protocols for the wound healing assay and spheroid formation can be found in *SI Appendix*.

### Statistical Analyses.

Statistical analyses and graphs were performed using GraphPad Prism® software (GraphPad Software, San Diego, CA). Values presented in the text and figures are as mean ± SEM from at least three independent experiments, except otherwise specified. The Student’s *t* test or the Mann–Whitney test were used to assess the statistical significance of differences between any two groups. Multiple quantitative datasets were compared by using one-way or two-way ANOVA with Tukey’s post hoc test. Differences with *P*-values less than 0.05 were considered statistically significant. *P*-values, statistical tests, and number of replicates (n) are indicated in *SI Appendix*, Table S3 for every single quantification presented in this manuscript.

### Molecular Docking and Dynamics Simulation.

Molecular docking and molecular dynamics simulation are described in *SI Appendix*.

### Development of LV80.

The steps leading to the development of EpoY derivatives, including LV43, LV80, and biotinylated LV80 are detailed in *SI Appendix*.

## Supplementary Material

Appendix 01 (PDF)

## Data Availability

All study data are included in the article and/or *SI Appendix*.
