# Supplementary material for: Peptide-based covalent inhibitor of tubulin detyrosination promotes mesenchymal-to-epithelial transition in lung cancer cells
Source: Proc Natl Acad Sci U S A. 2025 Dec 31;123(1):e2514990123. doi: 10.1073/pnas.2514990123 (PMC12773777; doi:10.1073/pnas.2514990123)
Supplement: Supplementary file 1 — Appendix 01 (PDF) [file pnas.2514990123.sapp.pdf]

PNAS

Supporting Information for
Peptide-based covalent inhibitor of tubulin detyrosination promotes mesenchymal-to-epithelial transition in lung cancer cells.

Hathaichanok Impheng[1†], Ghislain Gillard[2†], Nuttanid Numnoi[1], Anthony Feral[3], Matthieu Simon[3], Maxime Louet[3], Muriel Amblard[3], François Juge[2], Lubomir Vezenkov[3*], Krzysztof Rogowski[2*]
[†]These authors contributed equally.

* Lubomir Vezenkov, Krzysztof Rogowski
Email: lubomir.vezenkov@enscm.fr
krzysztof.rogowski@igh.cnrs.fr

This PDF file includes:

Supporting text for Materials and Methods
Figures S1 to S7
Tables S1 to S3
SI References

Supporting Information Text

Materials and Methods

Cell culture, generation of knockout lines and siRNA

Cell culture

A549 lung carcinoma, HEK293 kidney epithelial, CHL-1 skin melanoma, and normal lung IMR-90 fibroblast cells were cultured in appropriate growth media based on cell type. A549 cells were maintained in Dulbecco's Modified Eagle Medium (DMEM), HEK293 cells and CHL-1 cells in DMEM/F-12 GlutaMAX, and IMR-90 cells in Minimum Essential Medium (MEM) (all from Gibco). H520, H1975, and H1299 human lung cancer cells were cultured in RPMI-1640. The media were supplemented with 10% heat-inactivated fetal bovine serum (FBS) (Gibco) and 1% penicillin/streptomycin solution (Gibco) to ensure optimal growth conditions. All cells were incubated under a humidified atmosphere of 5% CO_2 at 37°C.

CRISPR/Cas9-based generation of knockout cell lines

A549 cells were transfected with plasmids encoding gRNAs (Sanger whole genome CRISPR library, Sigma) and Cas9 (pSpCas9(BB)-2A-GFP (PX458), Addgene #48138) at a 4:1 (gRNA:Cas9) ratio using jetPEI (Polyplus) according to manufacturer's protocol. The expression of BFP and GFP by the plasmids encoding the gRNA and the Cas9, respectively, allowed for fluorescent-based cell sorting of the double-transfected cells. 24 hours after transfection, cells were trypsinised and collected in DMEM-F12 for cell sorting of BFP- and GFP-double positive cells on a Cytek Aurora CS cell sorter (Institute for Regenerative Medicine and Biotherapy, Montpellier). Clones were grown on 10 cm plates by limited dilution. Clones were manually picked with paper soaked in trypsin and transferred into 24-well plates for characterization. Putative positive clones were first analyzed by immunofluorescence for their levels of detyrosination. Knockout clones were confirmed both by western blot analyses and genome sequencing of the proper locus (e.g. TTL, VASH1 and/or VASH2). Note that VASH1+2KO/TTL-KO #1 and #2 have been obtained by performing CRISPR-based knockout of TTL in VASH1+2KO, that is knockout for VASH1 and VASH2. The gRNA sequences used to generate these knockout cell lines are presented in Table S1. To identify the mutations generated in the VASH1 and VASH2 loci by the CRISPR strategy, genomic DNA from cultured cells was prepared using NucleoSpin Tissue kit from Macherey-Nagel. For amplification and sequencing of the TTL locus, we prepared cDNA from cultured cells using the Maxima First-strand cDNA synthesis kit (Thermo Fisher). The genomic region surrounding the CRISPR target site was amplified using the primers indicated in Table S2.
PCR fragments were purified from agarose gels (Macherey-Nagel Gel and PCR clean-up kit) and sequenced using nanopore sequencing by Smartlife Biosciences. Between one to five thousand sequences were aligned to a reference sequenced obtained from amplification of the same regions on wild-type cells to generate .bam files (Smartlife Biosciences). Alignments were manually analyzed using Geneious software to detect mutations around the CRISPR target site. TTL-KO cells were identified and validated by the lack of TTL expression using immunoblot analyses.

siRNA experiments

Knock-down of E-cadherin was performed with pre-designed Dicer-Substrate Short Interfering RNAs from Integrated DNA Technologies (TriFECTa® Kit from IDT), which were transfected into A549 cells with RNAiMAX (Invitrogen) according to manufacturer's guidelines with 10 nM final of siRNA. Cells were collected 48h post-transfection for biochemical and immunofluorescence analyses. Knock-down cells were further analyzed by wound healing assay and hanging drop analysis at indicated time points. References of the DsiRNA are hs.Ri.CDH1.13.1, hs.Ri.CDH1.13.2 and hs.Ri.CDH1.13.3.

Assessment of cell viability

The percentage of cell viability was determined from three independent experiments using the 3-(4,5-dimethylthiazol-2-yl)-5-(3-carboxymethoxyphenyl)-2-(4-sulfophenyl)-2H-tetrazolium (MTT) assay. Briefly, A549 and IMR-90 cells were seeded at densities of 15,000 and 20,000 cells per well,

respectively, and allowed to attach overnight before treatment with LV80, PTL and Taxol at the indicated concentrations. The duration of the treatment was 24h in CHL-1 cells and 48h for A549 and IMR90 cells. After the treatment period, the cells were washed with PBS and then incubated with MTT solution (5 mg/mL in PBS) for 2 hours at 37°C. The resulting formazan crystals were dissolved in DMSO, and the absorbance was measured at 595 nm using a Synergy HT Microplate Reader (BioTek Instruments, Inc, Winooski, VT, USA).

Incubation with drugs and inhibitors

Experimental design for cell treatments with various drugs and inhibitors

For the *in cellulo* characterization of the different inhibitors (EpoY, LV43, LV80, biotinylated LV80), cells were pre-incubated with the inhibitors at the indicated concentrations for 3h followed by the addition of 10 µM Taxol and subsequent 45 min incubation prior to collection and immunoblot analysis. Due to a strong lethality induced by parthenolide treatment, in the experiment presented in Fig. 1C the pre-treatment was omitted and the inhibitors EpoY and PTL were added concomitantly with Taxol.

Immunoblots, immunofluorescence or phenotypic analyses (cell scratching and hanging drop assays) of the inhibition of endogenous detyrosination were performed in the presence of 100 µM of LV80 for either 48h in the case of A549, CHL-1 and H1299 cells or 24h for H1975 and H520 cells. Importantly, no additional LV80 was added to the cells during the course of wound closure and 3D spheroid formation experiments. Similarly, A549 TTL-KO cells were treated with LV80 for 48h to investigate E-cad protein levels. E-cad protein degradation was assessed by incubating A549 cells with 50 µg/mL of cycloheximide (Sigma, 239763-M) for 9, 24 or 48h prior to collection for immunoblot analysis. Bafilomycin A1 (Sigma, B1793) was incubated for 4h at 100 nM final.

IC_{50} calculations

For the IC_{50} calculations the levels of detyrosinated tubulin were quantified using immunoblot and normalized with α-tubulin serving as an internal control. For each concentration, the normalized level of detyrosinated tubulin was expressed as a percentage of relative activity with respect to the Taxol-induced condition in the absence of the inhibitor (set to 100%). Inhibitor concentrations were Log10-transformed and the dose-response relationship was fitted by nonlinear regression using a four-parameter logistic (variable-slope) model to estimate the IC_{50} (reported in µM) and its 95% confidence interval. The quantification was performed with data derived from three independent experiments.

Immunofluorescence analysis

Staining, imaging and data integration

Monolayer A549, dropped A549 (4000 cells per 10 µL media drops) and CHL-1 were fixed with cold methanol for 5 min at –20°C. Immunostainings were performed in PBS supplemented with 3% BSA and 0.1% Triton. Primary antibodies used were mouse anti-E-cadherin (BD Transduction Laboratories, 610181) 1/1000, mouse DM1A (α-tubulin, Sigma, T9026) 1/1000, rabbit anti-c-myc (Abcam, Ab32072) 1/500, rabbit anti-non phosphorylated (active) β-catenin (Cell Signaling, 8814) 1/500, rabbit anti-Cyclin D1 (Cell Signaling, 2978) 1/500, mouse anti β-catenin (BD Transduction Laboratories™, 610154) 1/500, rabbit anti-Δ1-tubulin 1/1000 (clone RM444, RevMAb Biosciences, 31-1335-00), rabbit anti-Lamp1 (Sigma, L1418) 1/2000, mouse anti-centrin (Sigma, 04-1624) 1/1000, rabbit anti-Vimentin (Cell Signaling, 5741) 1/1000, Rat YL1/2 (anti-Tyr-Tub) 1/1000 (1) . Secondary antibodies coupled to Alexa-488 (A-11008), Alexa-555 (A-31570) or Alexa-647 (A78947) from Invitrogen Molecular Probes were used at 1/1000. Acquisitions were performed as 0.5 µm-spaced optical sections with a 63X Plan Apochromat 1.4 NA oil DIC objective on a Leica SP8 confocal equipped with hybrid detectors with a GaAsP photocathode. Z-stack reconstructions were performed in Fiji (2) and triple stainings were color-coded using the BOP LUTs kindly made available by Christophe Leterrier (INP, Marseille).

Fluorescence intensity quantifications

For c-myc and Cyclin D1 intensity, a mask was created on the DAPI channel using the Otsu thresholding method followed by a watershed to separate touching objects. Fluorescence intensity was quantified for particles over 50 pixels in size and normalized over the background of the field of view (average signal of 5 ROIs outside cell nuclei). β-catenin and non-phosphorylated β-catenin mean intensity values were measured after drawing the cell outlines (7-pixel wide line) with a home-made plugin in Fiji (3).

Immunoblot analysis

Immunoblot analysis SDS-PAGE was performed as previously described to separate α- and β-tubulin (4). Proteins were transferred onto 0.45 µm nitrocellulose membranes (Amersham), blocked with 5% milk in TBS-Tween20 followed by immunodetection. Membranes were incubated with rabbit anti-detyrosinated tubulin 1/1000 (clone RM444, RevMAb Biosciences, 31-1335-00), mouse 6-11B-1 (anti-acetylated tubulin, Sigma, T7451) 1/2000, mouse 12G10 (anti-α-tubulin, DSHB) 1/1000, rat anti-tyrosinated tubulin (YL1/2) 1/1000 (1), rabbit anti-Vimentin (Cell Signaling, 5741) 1/4000, mouse anti-E-cadherin (BD Transduction Laboratories, 610181) 1/1000, rabbit anti-SVBP (Sigma, HPA008507) 1/1000, mouse anti-Vinculin (Sigma, V9131) 1/1000, rabbit anti-GFP (Torey Pines Laboratories, TP401) 1/500, mouse GT335 1/1000 (5), polyE (Adipogen, AG-25B-0030-C050) 1/1000, mouse anti β-catenin (BD Transduction Laboratories™, 610154) 1/1000, rabbit anti-TTL (Proteintech, 13618-1-AP) 1/1000, rabbit anti-TMCP1 (Invitrogen, PA5-64255) 1/1000, mouse anti-VASH1 (Sigma, MABC537) 1/1000, mouse anti-N-cad (Affinity Biosciences, AF6710) 1/1000 mouse anti-p62 (Santa Cruz, sc-28359) 1/1000 and rabbit anti-LC3B (Sigma L7543) 1/1000 antibodies. Protein bands were visualized with HRP-labelled goat anti-rabbit (Cell Signaling), anti-mouse (Cell Signaling) or anti-rat IgG (Merck) all at 1/5000 followed by detection with chemiluminescence (SuperSignal West Pico PLUS, Thermo Scientific) using a Chemidoc Touch imaging system (Biorad) or chemiluminescence films (GE Healthcare). The levels of detyrosinated tubulin in A549 cells cultured in hanging drops were further quantified at 24, 48, and 72 hours across three independent experiments. A549 cells (4,000 cells per 10 µL media drop) were placed on the lid of a 6 cm plate filled with PBS and cultured for the indicated time period. After the incubation, the cells were collected (total of 40 drops per set) and lysed using a lysis buffer containing beta-mercaptoethanol, followed by boiling at 95°C for 5 minutes. The boiled protein samples were then separated by SDS-PAGE, as previously described. The relative expression levels of detyrosinated tubulin, E-cadherin, N-cadherin and vimentin were quantified and normalized over α-tubulin using ImageJ and Image Lab (Biorad) softwares. In order to assess E-cad degradation over time in CHX experiments, the ratio of E-cad/Tubulin for each time point were normalized to the T0 time point of the corresponding genotype that was set at 100%.

Droplet digital PCR

To quantify expression levels in wild type, knockout or LV80-treated cells, we prepared cDNAs using the Maxima First-strand cDNA synthesis kit (Thermo Fisher). Expression levels were compared to the reference gene TBP by multiplex droplet digital PCR (ddPCR) using fluorescent probes sets. Mixes of primers and probes for each target gene were purchased from IDT: TBP ref. #Hs.PT.58v.39858774, VASH1 ref. Hs.PT.58.40217035, VASH2 ref. #Hs.PT.58.38647032, E-cadherin ref. #Hs.PT.58.3324071. Multiplexed PCR were performed using ddPCR Supermix for Probes (Bio-Rad) and analyzed with QX600 Droplet Digital PCR system (Bio-Rad).

Biochemistry

<u>Expression and purification of recombinant proteins</u>
Human TMCP1 ORF was cloned into pET28A+ to produce His-hTMCP1 as previously described (6) . To obtain His-hVASH1/hSVBP and His-hVASH2/hSVBP complexes, two bicistronic vectors were constructed. VASH1 and VASH2 coding sequences were first cloned into pET28a+ vector using BamHI and EcoRI sites to produce His-tagged VASH1 and VASH2. Then a DNA fragment (synthetized by Integrated DNA technologies, Inc.) containing a ribosome-binding site from T7 phage gene 10 and the T7 tag in frame with SVBP was cloned downstream from VASH1 or VASH2

in pET28a using HindII and XhoI sites. The sequence of the T7-SVBP fragment is: 5'-TTCTAAGCTTGAAATAATTTTGTTTAACTTTAAGAAGGAGATATACCATGGCTAGTATGACT
GGTGGACAGCAAATGGGTCGCACTAGTATGGACCCACCTGCACGTAAAGAAAAAACCAAAG
TTAAAGAATCTGTCAGCAGAGTTGAGAAGGCCAAACAGAAATCAGCCCAGCAGGAGCTGAA
GCAGAGACAAAGAGCAGAGATTTATGCCCTCAACAGAGTCATGACAGAACTGGAGCAGCAG
CAGTTTGATGAGTTCTGTAAACAGATGCAGCCTCCTGGAGAATGACGTCGCGGCCGCACTC
GAGCACC-3'. BL21 Star (DE3) bacteria transformed with the appropriate vector were grown at 37°C until the optical density (OD600) reached 0.6 then induced with 0.5 mM isopropyl-β-d-thiogalactoside (IPTG) overnight at 18°C. Bacteria were collected and disrupted using a HTU-DIGI-F press (Heinemann). Recombinant proteins were purified using nickel-based affinity chromatography (IMAC) according to the manufacturer's protocol (GE Healthcare).

Labelling of GFP-VASH1 and GFP-VASH2 with biotinylated LV80

HEK293 cells were co-transfected with GFP-VASH1 or GFP-VASH2 and HA-SVBP for 24hrs. Following transfection, cells were treated overnight with either DMSO or 50 µM of biotinylated LV80. Cells expressing GFP-VASH1 and GFP-VASH2 treated with either DMSO or biotinylated LV80 were lysed in PBS supplemented with 0.2% NP-40 and immunoprecipitated using GFP-trap (Proteintech, gtma) according to the manufacturer protocol. The precipitated proteins were subjected to immunoblot analysis and the biotinylated VASH1 and VASH2 were revealed with neutravidin HRP (Thermo Scientific, 31001).

Biotinylated-LV80-dependent precipitation of endogenous VASH1 from either HEK293 cells or mouse brain extract.

HEK293 cells or mouse brain were lysed in 50mM Tris pH7.5, 250mM NaCl and 1% Triton X100. The corresponding extracts were supplemented either with DMSO or 50 µM of biotinylated LV80 and incubated for 1hr at 4°C followed by the addition of Dynabeads MyOne Streptavidin C1 (ThermoFisher Scientific, #65001) for an additional 1h incubation at 4°C. The pull-down proteins were subjected to immunoblot analysis.

In vitro detyrosination assay

Sf9 cells were grown, lysed and used for tubulin purification by affinity chromatography, as previously described (7). To prepare MTs, purified tubulin was resuspended in 80mM PIPES pH 6.8 and 10% glycerol and incubated at 37°C for 30 min in the presence of 1mM GTP and 20 µM Taxol. After polymerization, MTs were pelleted by ultracentrifugation at 100,000g for 30 min at 37°C, resuspended in 80 mM PIPES pH 6.8, 10% glycerol with 20 µM Taxol, snap frozen and stored at -80°C in aliquots. *In vitro* analysis of detyrosination activity was performed in the presence of 20 µM Taxol using the recombinant hVASH1/SVBP or hVASH2/SVBP at 250 nM. Detyrosination assays were performed in 50 mM sodium phosphate buffer in the presence of 4 µg of MTs polymerised from Sf9 tubulin for 1 hour at 37°C. The concentration of inhibitors used in these *in vitro* assays (EpoY, Sigma, SML2301; parthenolide, Sigma, P0667; LV43 and LV80, this study) are indicated in the figures. Benzylsuccinic acid (BzISA) was used as a positive control inhibitor of CPA (Sigma, C9268) activity. Samples were mixed with loading buffer, boiled for 5 min at 95°C and subjected to immunoblotting. Quantifications were performed using ImageJ software. His-hTMCP1 was incubated at a final concentration of 250 nM with 4 µg of Sf9 MTs as previously described (6) in the presence of increasing concentrations of LV80. EDTA (2 mM final concentration) was used as a positive control inhibitor of TMCP1 activity.

Deglutamylation assay

Deglutamylation assays have been performed as previously described (8). Briefly, extracts from CCP-expressing HEK293 cells were incubated for 2 hours at 37°C with either the brain tubulin or tubulin purified from HEK cells transfected with TTLL4 monoglutamylase. Enzymatic activities were determined by running the samples on SDS-PAGE and immunoblot analysis.

Wound healing and hanging drop assays

Wound healing assay

A549 cells were seeded at a density of 1.5 × 10^5 cells per well in a 24-well plate. After 24 hours, when cells reached approximately 90% confluency, a wound was created by scratching the monolayer with a sterile 200 µL pipette tip. Detached cells were then carefully washed away with PBS, and the remaining cells were incubated in fresh medium containing 0.1% FBS, with or without the LV80 inhibitor. Wound closure was monitored and captured at 0, 24, and 48 hours using an inverted optical microscope (IX71, Olympus, Japan) equipped with a digital camera, using a 4X objective lens. The percentage of wound closure at each time point was quantified by normalizing the wound area to the 0-hour time point, using ImageJ software.

Hanging drop assay – Spheroid formation

A549 cells were prepared as a single-cell suspension in complete DMEM with or without the LV80 inhibitor. Drops of 10 µL, each containing 4,000 cells, were carefully placed on the lid of a 6 cm cell culture dish, which was filled with PBS at the bottom to maintain humidity. The cells were cultured at 37°C in a 5% CO_2 incubator. Spheroid formation was monitored and captured at specified time points (0, 24, 48, and 72 hours) using an Olympus BX53 F2 biological microscope equipped with 4X and 10X objectives. The percentage of inhibition of spheroid formation was calculated by comparing the area of spheroids in treated samples to the area of spheroids in untreated wild-type (WT) controls at 48 or 72 hours, using ImageJ software.

Molecular docking and dynamics simulation

Molecular Docking

Molecular Docking with Autodock4.6 (9) was used to predict the binding poses of the trans epoxy succinate (TES)-tyrosine (named EpoY) and the lead compounds LV43 and LV80. The protonation states and the generation of 3D conformations of ligands were generated by MarvinSketch®. The structure of the target was retrieved from the Protein Data Bank (PDB), and chosen due to the presence of TES-tyrosine covalent inhibitor, similar to the synthetized derivatives (PDB id: 6J7B) (10) . The protonation state of the target was predicted with Propka (11). The ligands and target were prepared using Autodock Tools (9). During the docking procedure, atoms of the protein were rigid while all the rotatable dihedral angles of the compounds were set flexible as in typical Autodock4 protocols. Residues G1 to F4 were removed and not considered in the docking procedure, due to their high flexibility. The flexibility of the N-terminal region was assessed by Molecular Dynamics (MD) simulations (Fig. S1G), which was confirmed by the lack of structural information in all other experimental structures of VASH1 in the PDB. The box size for grid mapping was set to 20.25 Å, 33.0 Å and 23.25 Å in x, y and z direction respectively, and centered near F146 and R166 to encompass largely the binding cleft (Fig. S1H). The grid spacing was kept to its default value (0.375 Å). In order to restraint the conformational space, we used a covalent docking procedure. For each compound, the carbon covalently bound to C169 of VASH1 was chosen accordingly to the X-ray structure of VASH and EpoY with a well of 3 Å wide and an energy barrier of 10 kcal/mol nearby the sulfur atom of C169. We generated 200 poses for each ligand-target couples using the genetic algorithm with 27000 generations, a population size of 300 and 50000000 energy evaluations. Finally, the poses were clustered according to their RMSD and the best poses in term of binding free energy (score) were further analyzed.

Molecular Dynamics Simulation

Molecular Dynamics (MD) simulations were conducted to explore the dynamical behavior of the catalytic site of VASH1. The crystallographic structure of VASH1 bound to epoxy succinate (TES)-tyrosine was used as the starting point (PDB id: 6J7B) (9). The parameters for the covalently bound ligand were derived from the Charmm General Force Field (CgenFF) (12–14) while we used the CHARMM36m (15) parameters for ions, solvent and protein. The system was built using the CHARMM-GUI web-server (16, 17). The protonation state of the protein was predicted with Propka (11). All protein residues and termini were found in their canonical state at pH 7. The protein was solvated in a cubic water box which size was 97 Å in each direction. 76 sodium and 85 chloride

ions were added to the system to reach a concentration of 0.15 M and to neutralize the system, resulting in a system of 85684 atoms. The system was simulated with Gromacs 2019.1 (18). The CHARMM-GUI protocol was used to equilibrate the system in the NVT ensemble, decreasing the constraints on the protein to allow its smooth relaxation at 303.15 K. The system was then simulated in the NPT ensemble for production, using Nose-Hoover algorithm for temperature control (303.15 K) and Parrinello-Rahman algorithm for pressure coupling (1 bar). The covalent bonds involving hydrogens were set rigid with the LINCS algorithm as implemented in Gromacs (19) to use an integration step of 2 fs. The cutoff for the direct non-bonded interactions was set to 12 Å while we used a switching function to smooth the potential in the range 10-12 Å. The Particle Mesh Ewald (PME) algorithm was used to treat long-range electrostatic interactions, together with Periodic Boundary Conditions (PBC). The production was stopped after 200 ns after verifying the protein was stable and reached an equilibrium. The RMSD at the end of the simulation was of 2.5 Å away from its crystallographic state which is in the range of the resolution of the crystallographic structure (2.3 Å).

Development of LV80

Inhibitor synthesis

All fluorenylmethyloxycarbonyl (Fmoc) protected amino acids and 1-[bis(dimethylamino)methylene]-1H-1,2,3-triazolo[4,5-b]pyridinium3-oxid hexafluorophosphate (HATU) were provided by Iris Biotech GmbH. Piperidine, N,N-diisopropylethylamine (DIEA), trifluoroacetic acid (TFA), triisopropylsilane (TIS), dichloromethane (DCM), 1,2-dichloroethane (DCE), N,N-dimethylformamide (DMF), N-methylpyrrolidinone (NMP) and ethyl ether (EtOEt) were provided by Sigma Aldrich. AmphiSpheres 40 RAM 0.38 mmol/g 75-150 µm resin was purchased from Agilent Technologies. Solvents used for HPLC and LC/MS were of HPLC grade. All final compounds were purified by reversed-phase 18 HPLC and the purity assessed by analytical reversed-phase HPLC.

SPPS procedure

Peptide synthesis was performed using a standard SPPS protocol. Each synthesis was performed using Fmoc-Rink amide AmphiSpheres 40 resin (0.37 mmol/g). The Fmoc protected amino acids (4 eq), HATU (4 eq) and DIEA (6 eq) were added to the syringe reactor and the mixture was stirred at room temperature (RT) for 1 hour. After each coupling reaction, the peptide-resin underwent two 5min deprotection cycles with DMF/piperidine 80/20 v/v solution.

Inhibitors' purification

All crude compounds were purified by preparative HPLC (Waters 4000 apparatus) on a C18 reversed-phase column (C18 Deltapak column, 100 mm x 40 mm, 15 µm, 100 Å) at a flow rate of 50 mL/min of a H2O + 0.1% TFA and CH3CN + 0.1% TFA mixture in gradient mode with UV detection at 214 nm. Fractions containing the pure product were collected and lyophilized.

LC/MS Analyses

Samples were prepared in an acetonitrile/water (50/50 v/v) mixture containing 0.1% TFA. The LC/MS system consisted of a Waters Alliance 2690 HPLC coupled to a Micromass (Manchester, UK) ZQ spectrometer (electrospray ionization mode, ESI+). All analyses were carried out using a C18 Chromolith Flash 25 × 4.6 mm column. A flow rate of 3 ml/min and a gradient of 0–100% acetonitrile over 5 min of H2O/0.1% HCOOH and CH3CN/0.1% HCOOH mixture in gradient mode with UV detection at 214 nm. Positive-ion electrospray mass spectra were acquired at a solvent flow rate of 100–200 µl/min. Nitrogen was used as nebulizing and drying gas. Data were obtained in scan mode, ranging from 200 to 1700 m/z in 0.1 s intervals; ten scans were summed to obtain the final spectrum. Retention times are given in minutes. Solvents used for HPLC and LC/MS were of HPLC grade.

Synthesis of **Compound 1**

(+,-)-trans-Oxirane-2,3-dicarboxylic acid (200mg, 1.52mmol, TCI chemicals) was dissolved in 5ml of NMP, followed by HCl.H-Tyr(OtBu)-OtBu (500mg, 1.52mmol) and DIEA (1.340ml, 8mmol). Then, PyAOP was added (792mg, 1.52mmol) and the reaction mixture was stirred at RT for 80min. The reaction mixture was then poured in 180ml of cold water. The obtained precipitate was removed by filtration. The pH of the filtrate was adjusted to 3 with 1M $KHSO_4$ solution and extracted three times with EtOAc. The organic phases were regrouped, washed with brine, dried over Na_2SO_4 and concentrated dry to yield 433mg of **Compound 1** as transparent oil. Expected mass: 618mg Obtained mass: 433mg Yield: 70% t_R = 3.4, MS (ESI+): m/z= 408.4 $[M+H]^+$

Synthesis of **LV43**

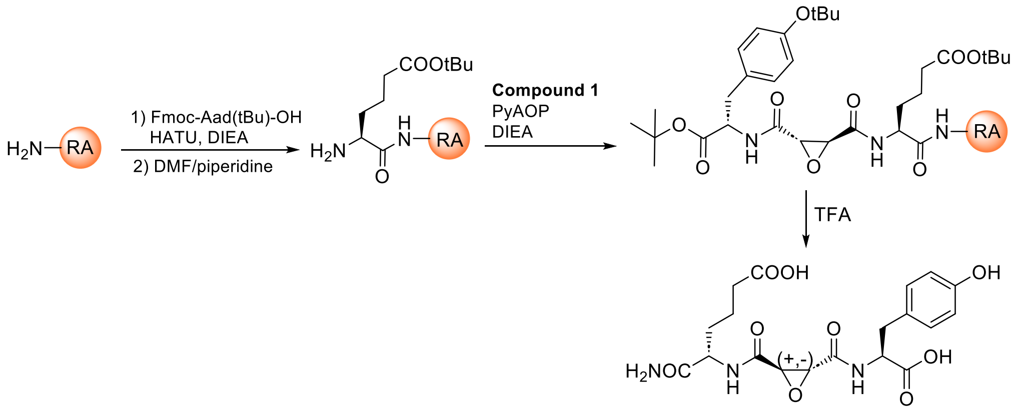

LV-43

Fmoc-Aad(OtBu)-OH (88mg, 0.216mmol) was attached to rink amide resin via a standard SPPS HATU (82mg, 0.216mmol), DIEA (50µl, 0.288mmol) activation. The reaction mixture was stirred with 200mg of ring amide resin charged at 0.37 mmol/g in the solid phase synthesis reactor for 2 hours. After standard washings, the Fmoc protection was removed by incubation in DMF/Pip 80/20 v/v for 10 min twice. Then, **Compound 1** (88mg, 0.216mmol) was attached to the liberated amine via PyAOP (113mg, 0.216mmol), DIEA (545 µl, 0.63mmol) activation. After overnight reaction, the resin was washed three times in DMF, three times in DCM, and the inhibitor was cleaved by incubation with 20 ml TFA for 50 min. The product containing the TFA filtrate was then evaporated dry, and the peptide was precipitated in EtOEt, filtered, purified by preparative HPLC, and lyophilized to obtain 10.7mg of **LV43** as a white solid.

Expected mass: 31.4mg

Obtained mass: 10.7mg

Yield: 34%

HPLC purity =100%, t_R = 0.84, MS (ESI+): m/z= 438,20 $[M+H]^+$ (expected m/z = 438.15)

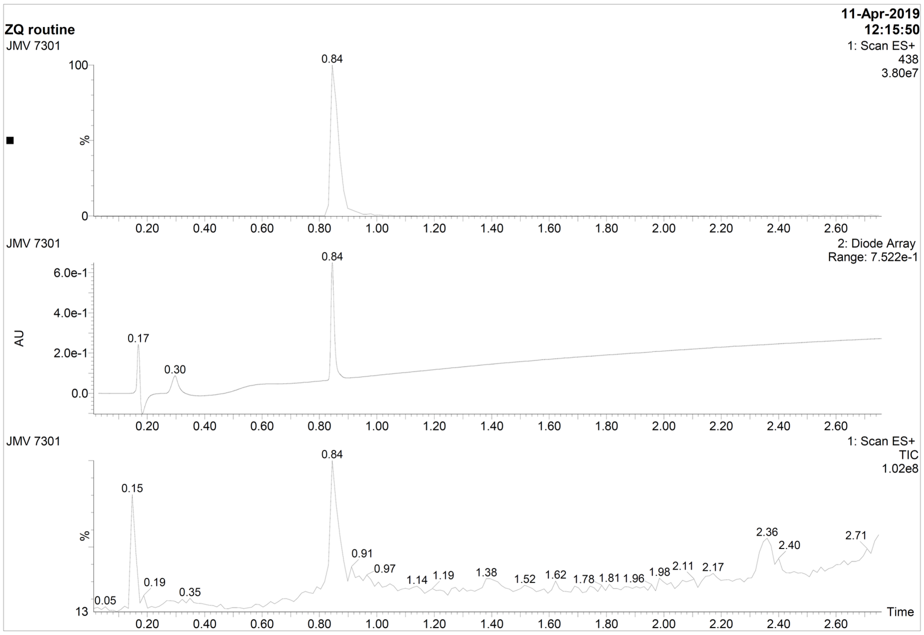

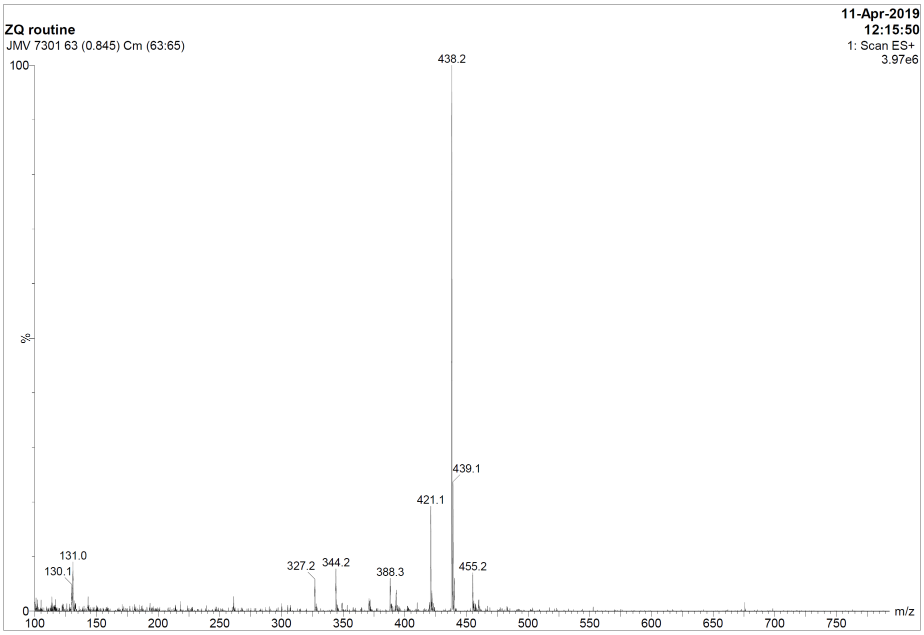

Synthesis of **Compound 2**

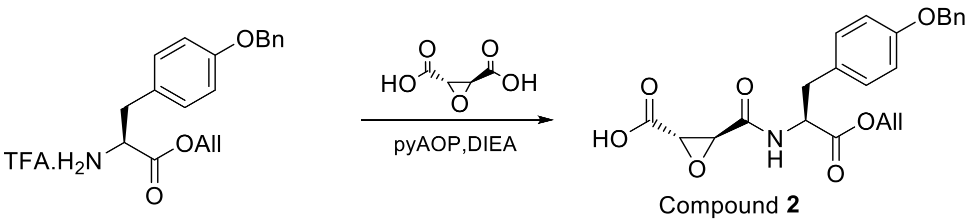

(2S,3S)-trans-Oxirane-2,3-dicarboxylic acid (66mg, 0.5mmol) was dissolved in 3ml of NMP, followed by the TFA.H-Tyr(OBn)-OAll (212mg, 0.5mmol) and DIEA (374µl, 2mmol). The RM was stirred and 260mg (0.5mmol) of PyAOP were added. The RM was stirred at room temperature for 20min and then poured in 50ml of cold water and kept in ice until precipitation appeared. The precipitate was removed by filtration and the filtrate pH was adjusted to 3 with 1M HCl solution. The

filtrate was then extracted 3 times with EtOAc and the organic phase was washed 3 times with 1M HCl solution, one time with brine, dried over $MgSO_4$ and evaporated dry to afford 100mg of **compound 2** as transparent oil. The product was used without further purification.
Expected mass: 212.5mg
Obtained mass: 100mg
Yield: 47%
HPLC purity = 95%, t_R = 3.33, MS (ESI+): m/z= 426,2 $[M+H]^+$, (expected m/z = 426.16)

<u>Synthesis of **Compound 3**</u>

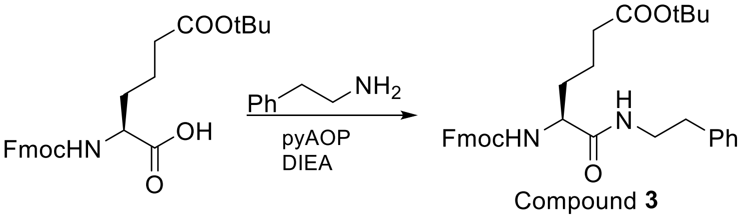

Compound **3**

Fmoc-Aad(OtBu)-OH (1g, 2.28mmol) was dissolved in 5ml of NMP followed by the 2-phenylethan-1-amine (316µl, 2.5mmol) and the DIEA (1.175ml, 6.83mmol). The RM was stirred and the PyAOP was added (1.18g, 2.28mmol). The RM was stirred for 15min at RT. It was then poured in 50ml of EtOAc, washed 1 time with water/brine 1/1 v/v mixture, 3 times $NaHCO_3$ saturated solution, 3 times 1M HCl solution, 1 time with brine, dried over $MgSO_4$ and concentrated dry to afford 1.24 g of **compound 3** as transparent oil. The compound was used without further purification.
Expected mass: 1.235g
Obtained mass: 1.24g
Yield: quantitative
HPLC purity = 95%, t_R = 4.28, MS (ESI+): m/z= 543,3 $[M+H]^+$

<u>Synthesis of **Compound 4**</u>

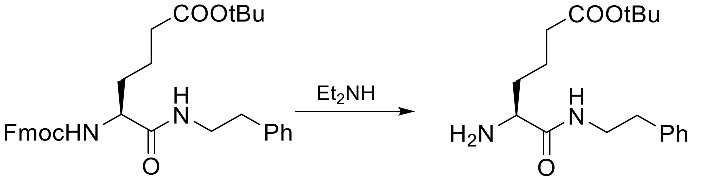

Compound **3** Compound **4** Yield 92%

Compound 3 (542mg, 1mmol) was dissolved in 5ml of NMP followed by the addition of diethylamine (208µl, 2mmol). The RM was then stirred at RT for one hour. Next, the RM was poured in 50ml of water and the pH was adjusted to 11 by NaOH 1M solution. The aqueous phase was then extracted 3 times with EtOAc, the regrouped organic phases were washed with brine, dried over Na_2SO_4 and concentrated dry to afford 294 mg of **Compound 4** as transparent oil. The product was used without any further purification.
Expected mass: 320mg
Obtained mass: 294mg
Yield: 92%
t_R = 2.28, MS (ESI+): m/z= 321.2 $[M+H]^+$

<u>Synthesis of **Compound 5**</u>

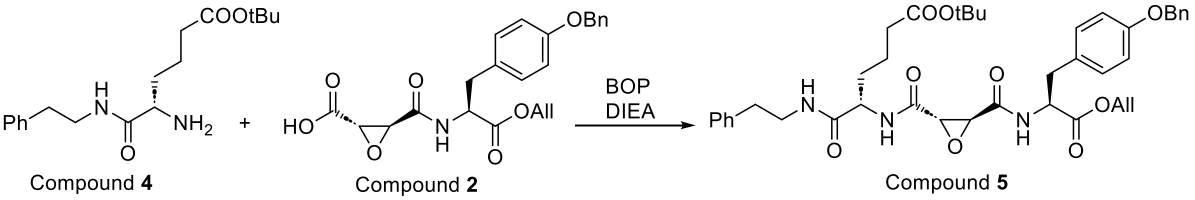

Compound **4** Compound **2** Compound **5**

Compound 4 (131mg, 0.41mmol) and **Compound 2** (174mg, 0.41mmol) were solubilized in 3ml DMF followed by the DIEA (139µl, 0.82mmol) and the BOP (173mg, 0.41mmol). The RM was stirred at RT for 2h and then an additional 51mg of BOP were added. After additional 30min, the RM was poured in 100ml of cold water. The obtained precipitate was filtered, washed 4 times with saturated $NaHCO_3$ solution, 4 times with 1M HCl solution and 4 times with water. The obtained white powder was then dried over night at 0.2mbar to afford 260mg of **compound 5** as white solid.
Expected mass: 260mg
Obtained mass: 300mg
Crude Yield: 86%

<u>Synthesis of **LV80**</u>

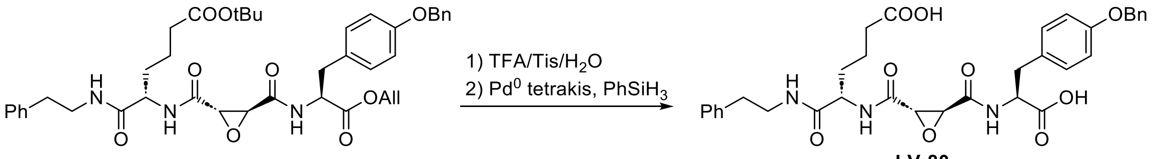

Compound 5 (100mg, 0.137mmol) was dissolved in 15ml of DCM, followed by 15ml of TFA/TiS/H_2O. The reaction mixture was then stirred at RT for 30min. The reaction mixture was concentrated dry and the remaining oil was precipitated with cold ether. The precipitate was filtered and dried over reduced pressure. Next, the precipitate was dissolved in 1ml of dry DMF and the allyl ester was removed by treatment with Pd^0 Tetrakis (5.15mg, 0.0045mmol), $PhSiH_3$ (37µl, 0.3mmol). After 20 min, the reaction mixture was dissolved in 20ml EtOAc that was washed once with 1M HCl solution, once with brine, and concentrated dry. The obtained precipitate was then purified by preparative HPLC to obtain 41mg of **LV80** as white solid.
Expected mass: 91mg
Obtained mass: 41mg
Crude Yield: 45%
t_R = 3.10, MS (ESI+): m/z= 632.4 $[M+H]^+$

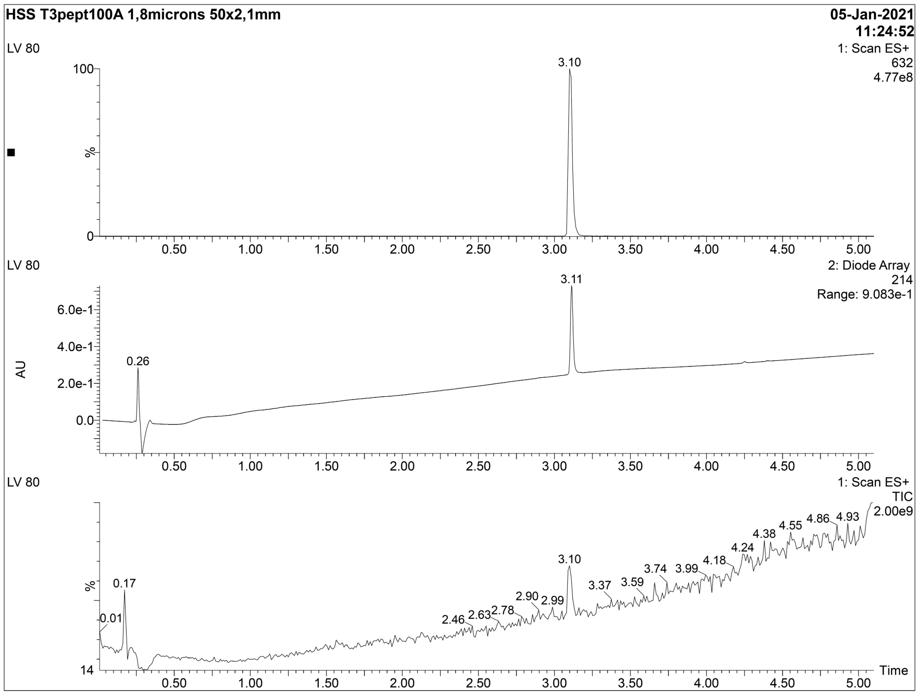

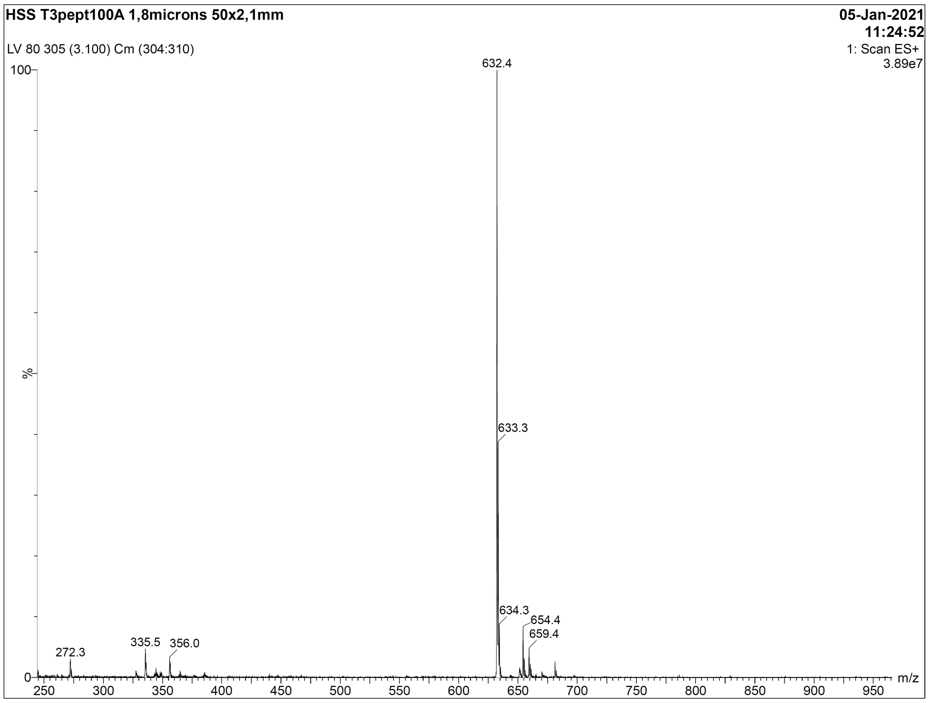

Synthesis of **Compound 6**

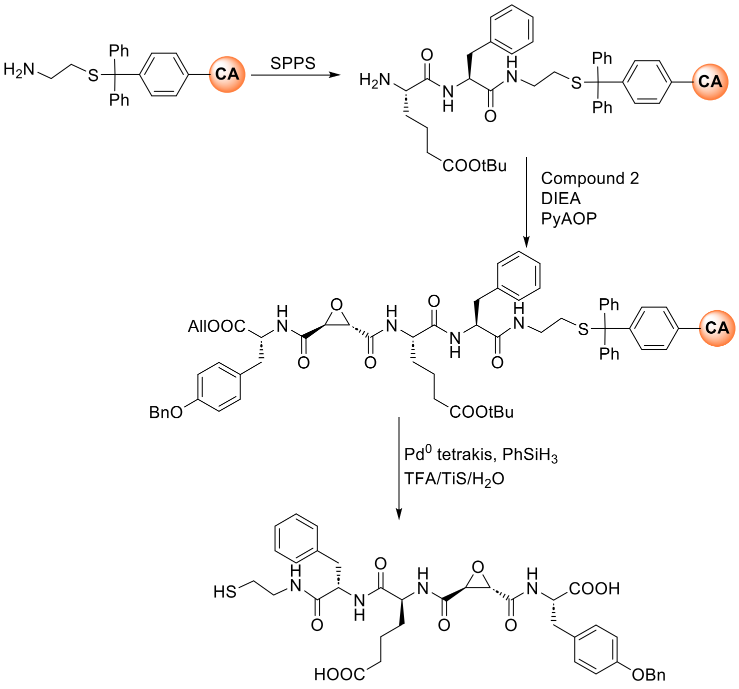

Compound **6**

The H-Aad(tBu)Phe- sequence was synthetized via SPPS on cystamine-Trt resin charged 0.5 mmol/g following standard SPPS. Next, the solid supported sequence was attached via an amide bond to **compound 2** (95mg, 0.225mmol) via PyAOP (95mg, 0.225mmol), DIEA (77µl, 0.450mmol) activation, overnight at RT. After a 2-hour long Pd Tetrakis (8.66mg, 0.008mmol), $PhSiH_3$ deprotection of the OAll ester in DCM the thiolated version of **LV80** was cleaved from the resin by 2-hour long treatment with TFA/DCM/H_2O/TiS 50/50/2.5/2.5. The filtrate was then concentrated dry

to provide a brown oil that was precipitated in cold EtOEt to afford 32mg of **compound 6** as white solid that. The compound was used without further purification.
Expected mass: 55mg
Obtained mass: 32mg
Crude Yield: 58%

Synthesis of Biotinylated **LV80** analogue

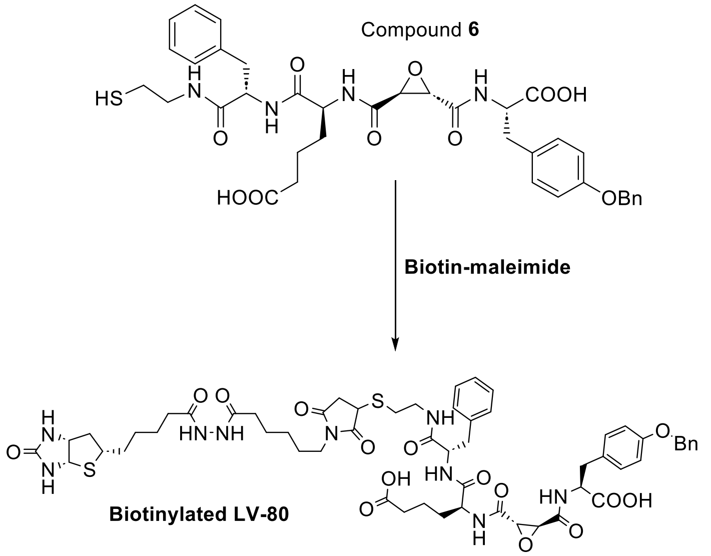

Compound 6 (7.35mg, 0.01mmol) was dissolved in 800µl of DMF followed by biotin-maleimide (4.5mg, 0.01mmol) and 400µl of PBS solution. The mixture was lightly heated to solubilize both reagents and then stirred at RT for 1h. The reaction mixture was then directly purified by preparative HPLC to obtain 7mg of **biotinylated LV80** as white solid.
Expected mass: 11.85mg
Obtained mass: 7mg
Yield: 59%
t_R = 2.89, MS (ESI+): m/z= 1186.2 $[M+H]^+$, 593.7 $[M+2H]^{2+}$

Figures

Fig. S1.

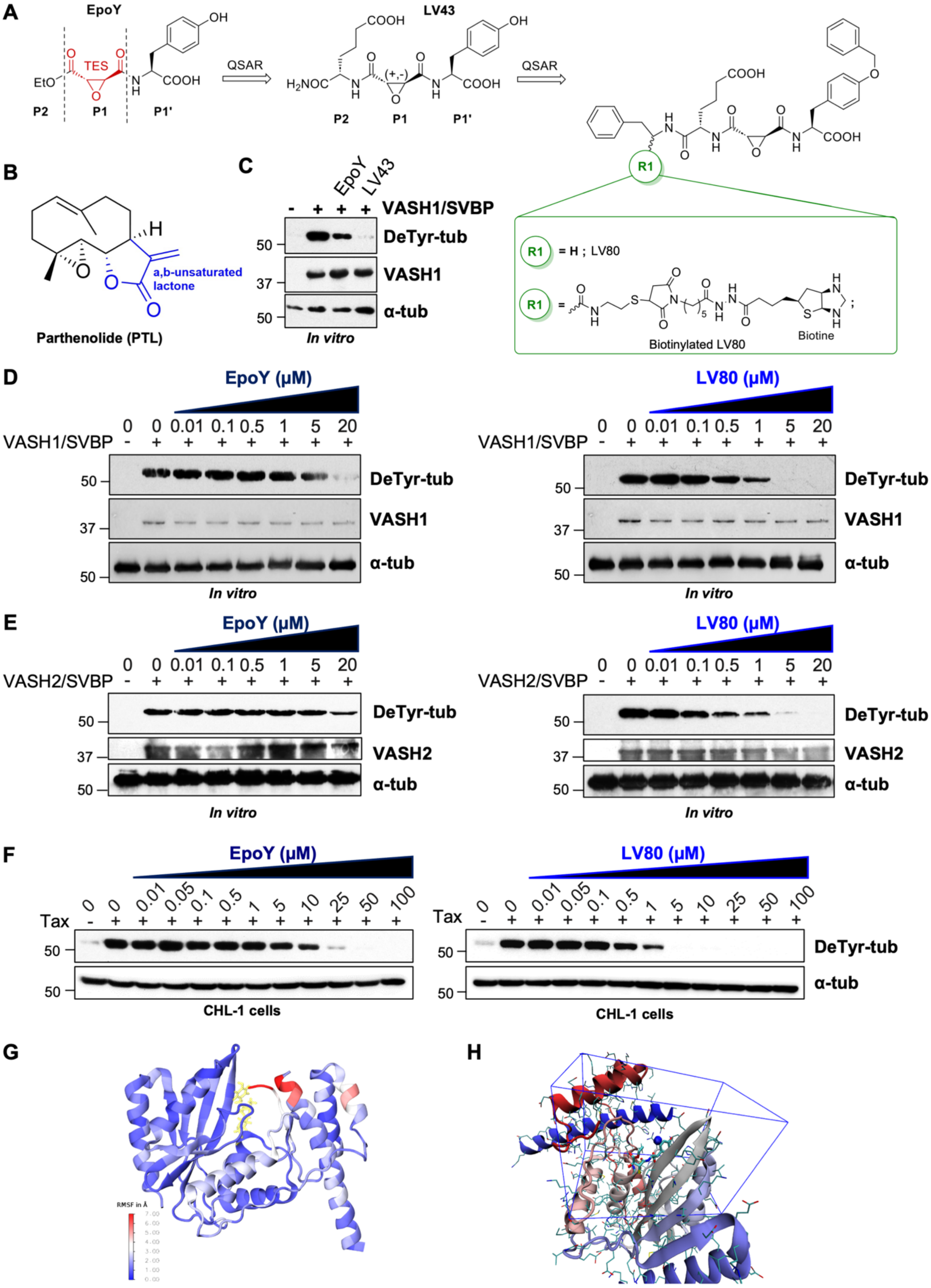

Development of LV80, a potent and specific VASH inhibitor (related to Figure 1). (**A**) Chemical structure of various epoxide-based inhibitors used in this study including EpoY, LV43, LV80 and its biotinylated version. (**B**) Chemical structure of parthenolide. The α, β-unsaturated lactone is highlighted in blue. (**C**) Immunoblot analysis of the *in vitro* assay involving recombinant VASH1/SVBP and Sf9-derived MTs comparing the inhibitory potency of EpoY and LV43. (**D**) Immunoblot analysis of the *in vitro* assay involving recombinant VASH1/SVBP and Sf9-derived MTs comparing increasing concentrations of EpoY and LV80. (**E**) Immunoblot analysis of the *in vitro* assay involving recombinant VASH2/SVBP and Sf9-derived MTs in the presence of increasing concentrations of EpoY and LV80. (**F**) Immunoblot analysis of the *in cellulo* assay involving CHL-1 cells treated with increasing concentrations of EpoY and LV80. (**G**) Root Mean Square Fluctuations (RMSF) of Cα of VASH1. The protein is represented as colored cartoon as a function of the value of the RMSF of each Cα in Å. The co-crystallized ligand (TES-tyrosine), present during the simulation has been drawn as transparent-yellow licorice. (**H**) Chosen box and its center for the molecular docking procedure. The boundaries of the searching box are represented by blue lines while its center is depicted as a blue sphere. The protein is represented in cartoon and colored according to the amino-acid indices (from red for the N-terminus to blue for the C-terminus). The side chains of the protein are represented by thin licorice and the co-crystallized ligand (EpoY) by thick licorice. Atoms are colored according to their atom types (carbon=cyan; nitrogen=blue; oxygen=red).

Fig. S2.

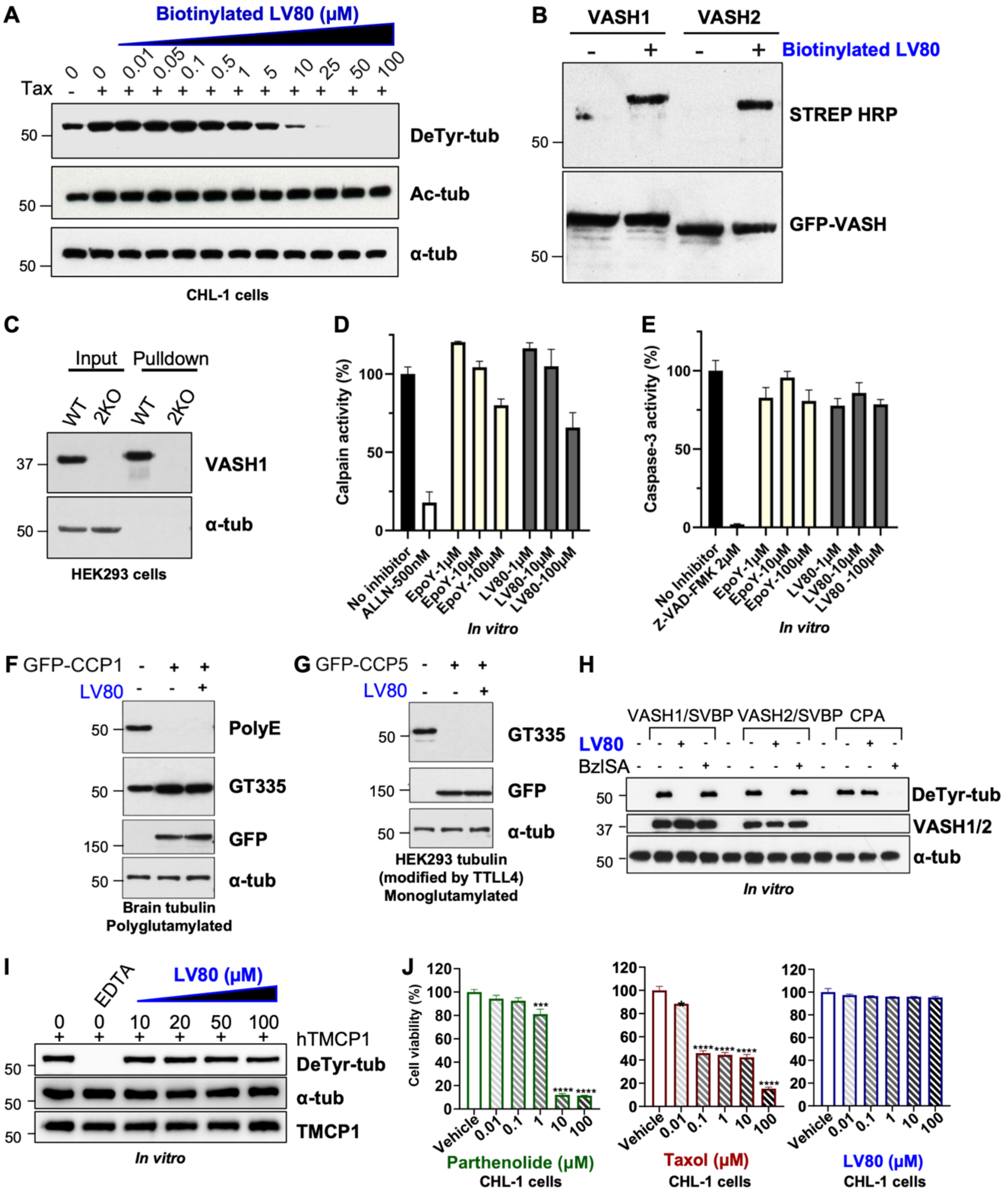

Characterization of LV80 inhibitory properties (related to Figure 2). (**A**) Immunoblot analysis of protein extracts from CHL-1 cells treated with increasing concentrations of biotinylated LV80 reveals a decrease in Taxol-induced detyrosination levels. (**B**) Immunoblot analysis of pull-down assays of GFP-VASH1 and GFP-VASH2 from HEK293 cells, in the presence of absence of biotinylated LV80. (**C**) Immunoblot analysis of the pull-down assay of endogenous VASH1 from HEK293 cell lysates using biotinylated LV80. Input and pull-down fractions were probed with indicated antibodies. (**D**) Quantification of calpain activity in the presence of increasing concentrations of EpoY and LV80. (mean +/- SEM, n=3 independent experiments). ALLN was used as a positive control for calpain inhibition. (**E**) Quantification of caspase-3 activity in the presence

of increasing concentrations of EpoY and LV80. Z-VAD-FMK was used as a positive control for caspase-3 inhibition. (**F**) Immunoblot analysis of an *in vitro* assay involving polyglutamylated brain microtubules treated with GFP-CCP1 deglutamylase in the presence or absence of LV80. (**G**) Immunoblot analysis of the *in vitro* assay involving MTs derived from HEK293 cells expressing TTLL4 monoglutamylase treated with GFP-CCP5 in the presence or absence of LV80. (**H**) Immunoblot analysis of the *in vitro* assay involving Sf9-derived MTs treated with VASH1/SVBP, VASH2/SVBP and CPA either alone, in the presence of LV80 or a CPA inhibitor called BzlSA. Please note that while LV80 efficiently inhibits VASH1 and VASH2, it has no effect on CPA activity. In contrast, BzlSA inhibits CPA but not VASH1 or VASH2 activity. (**I**) Immunoblot analysis of the *in vitro* assay involving recombinant human TMCP1 and Sf9-derived MTs in the presence of increasing concentrations of LV80. EDTA was used as a positive control for the inhibition of TMCP1 activity. (**J**) Graphical representation of the MTT assay involving CHL-1 cells treated with increasing concentrations of PTL, Taxol and LV80. In contrast to PTL and Taxol, the treatment with LV80 does not affect cell viability even at the highest concentration used.

Fig. S3.

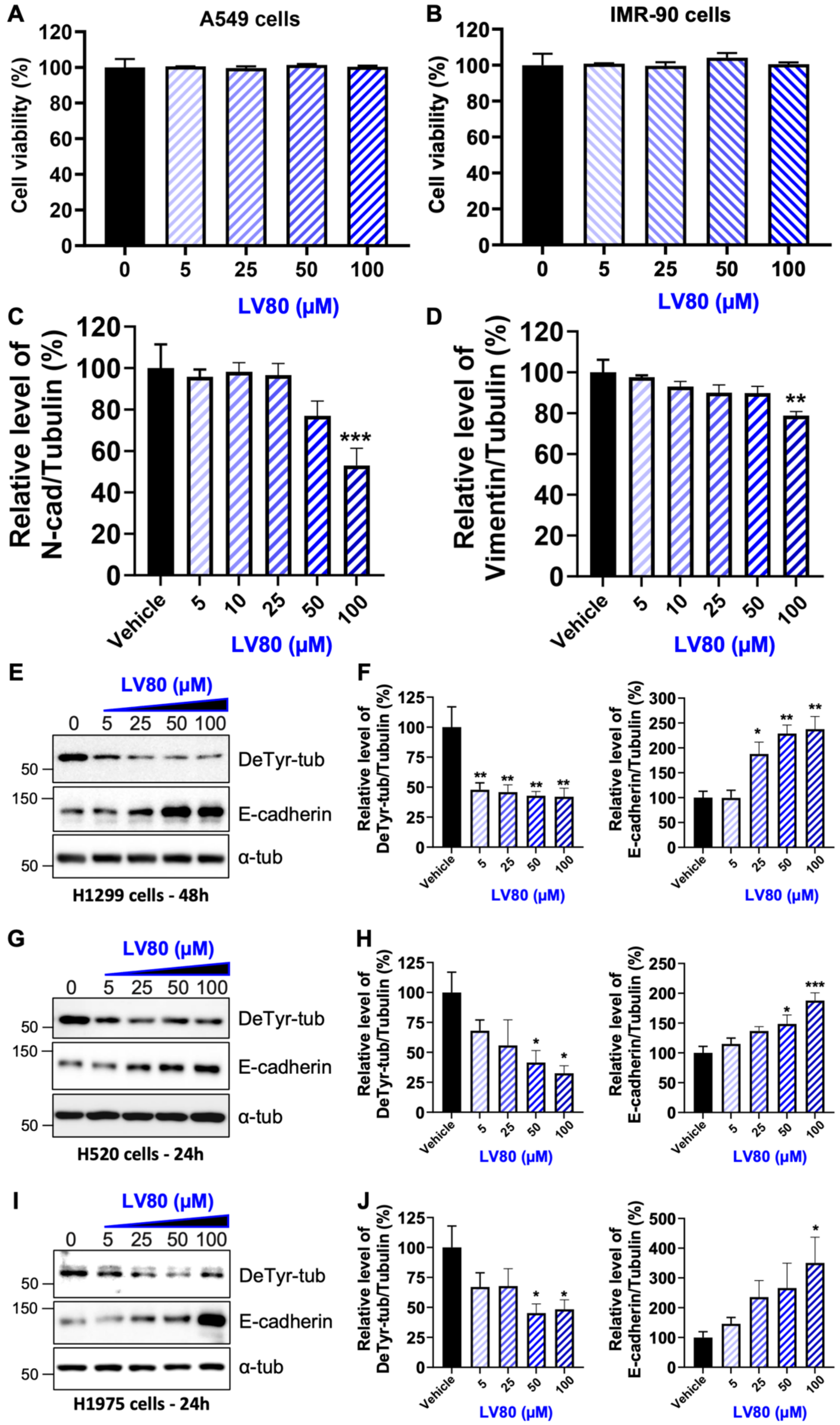
A
A549 cells
Cell viability (%)
LV80 (μM)
B
IMR-90 cells
Cell viability (%)
LV80 (μM)
C
Relative level of N-cad/Tubulin (%)
***
Vehicle
LV80 (μM)
D
Relative level of Vimentin/Tubulin (%)
**
Vehicle
LV80 (μM)
E
LV80 (μM)
DeTyr-tub
E-cadherin
α-tub
H1299 cells - 48h
F
Relative level of DeTyr-tub/Tubulin (%)
Relative level of E-cadherin/Tubulin (%)
LV80 (μM)
G
LV80 (μM)
DeTyr-tub
E-cadherin
α-tub
H520 cells - 24h
H
Relative level of DeTyr-tub/Tubulin (%)
Relative level of E-cadherin/Tubulin (%)
LV80 (μM)
I
LV80 (μM)
DeTyr-tub
E-cadherin
α-tub
H1975 cells - 24h
J
Relative level of DeTyr-tub/Tubulin (%)
Relative level of E-cadherin/Tubulin (%)
LV80 (μM)

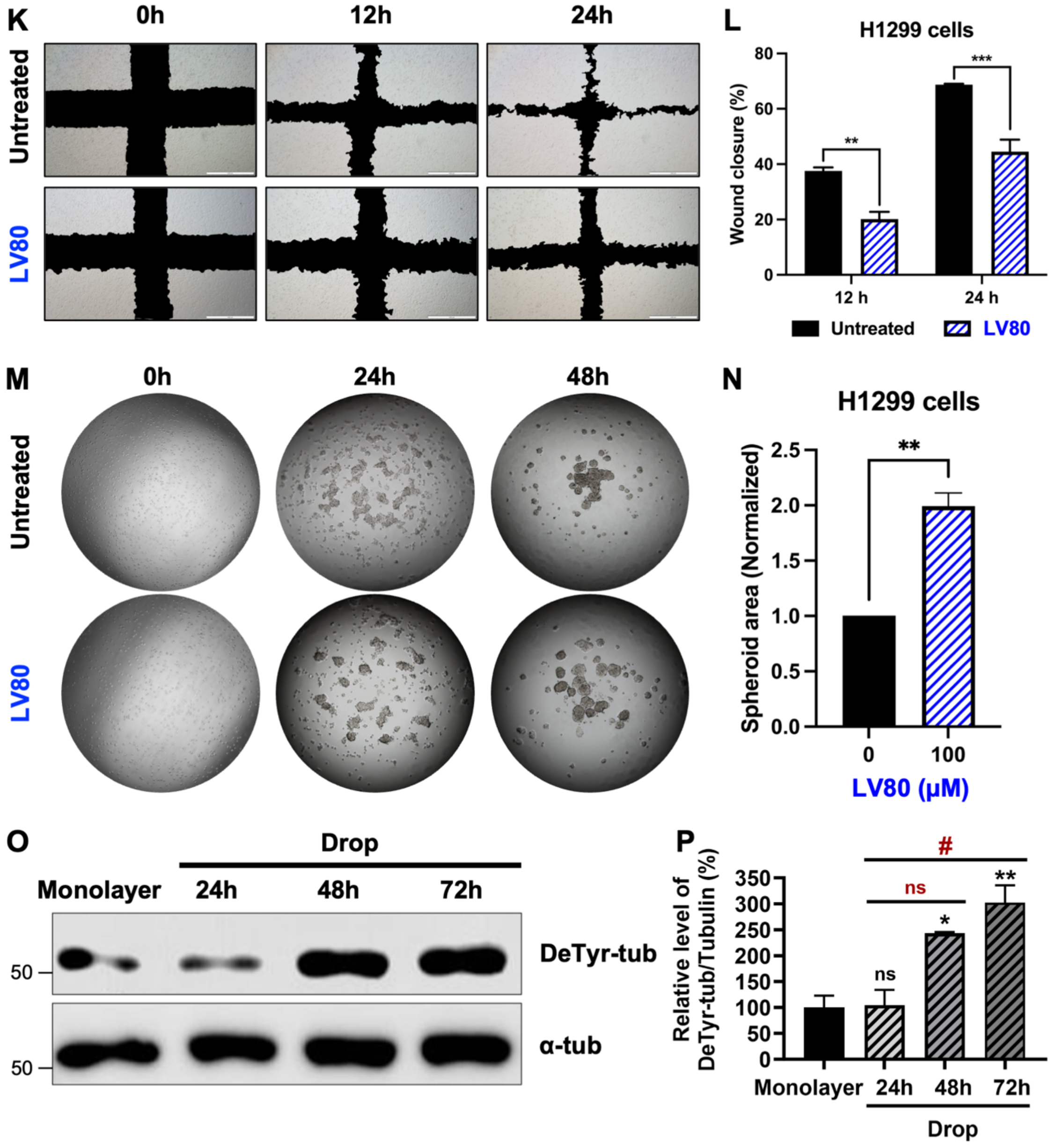

Role of tubulin detyrosination in the maintenance of the mesenchymal state (related to Figure 3). (**A**) Graphical representation of the MTT assays involving A549 cells treated with increasing concentrations of LV80 for 48h. Note the lack of toxicity even at the highest concentration. (**B**) Graphical representation of the MTT assays involving IMR-90 cells treated with increasing concentrations of LV80 for 48h. Note the lack of toxicity even at the highest concentration. (**C**) Quantification of N-cad protein levels in A549 cells treated with increasing concentrations of LV80. (**D**) Quantification of vimentin protein levels in A549 cells treated with increasing concentrations of LV80. (**E**) Immunoblots of protein extracts from H1299 cells treated with increasing concentrations of LV80 showing an increase in the levels of E-cad. (**F**) Quantification of detyrosination (left panel) and E-cadherin (right panel) levels in H1299 cells. (**G**) Immunoblots of protein extracts from H520 cells treated with increasing concentrations of LV80 showing an increase in the levels of E-cad. (**H**) Quantification of detyrosination (left panel) and E-cadherin (right panel) levels in H520 cells. (**I**) Immunoblots of protein extracts from H1975 cells treated with increasing concentrations of LV80 showing an increase in the levels of E-cad. (**J**) Quantification of detyrosination (left panel) and E-cadherin (ight panel) levels in H1975 cells. (**K**) Treatment of H1299 cells with LV80 affects cell migration as revealed by wound healing assay. (**L**)

Quantification of the wound closure percentage in WT or LV80-treated H1299 cells. (**M**) LV80 treatment prevents spheroid formation in H1299 cells. (**N**) Quantification of the normalized spheroid area after 48h of treatment with LV80. (**O**) Immunoblot analysis of protein extracts from A549 cells grown either on cell culture plates or in hanging drops for 24, 48 or 72 hours. (**P**) Quantifications of detyrosinated tubulin levels.

Fig. S4.

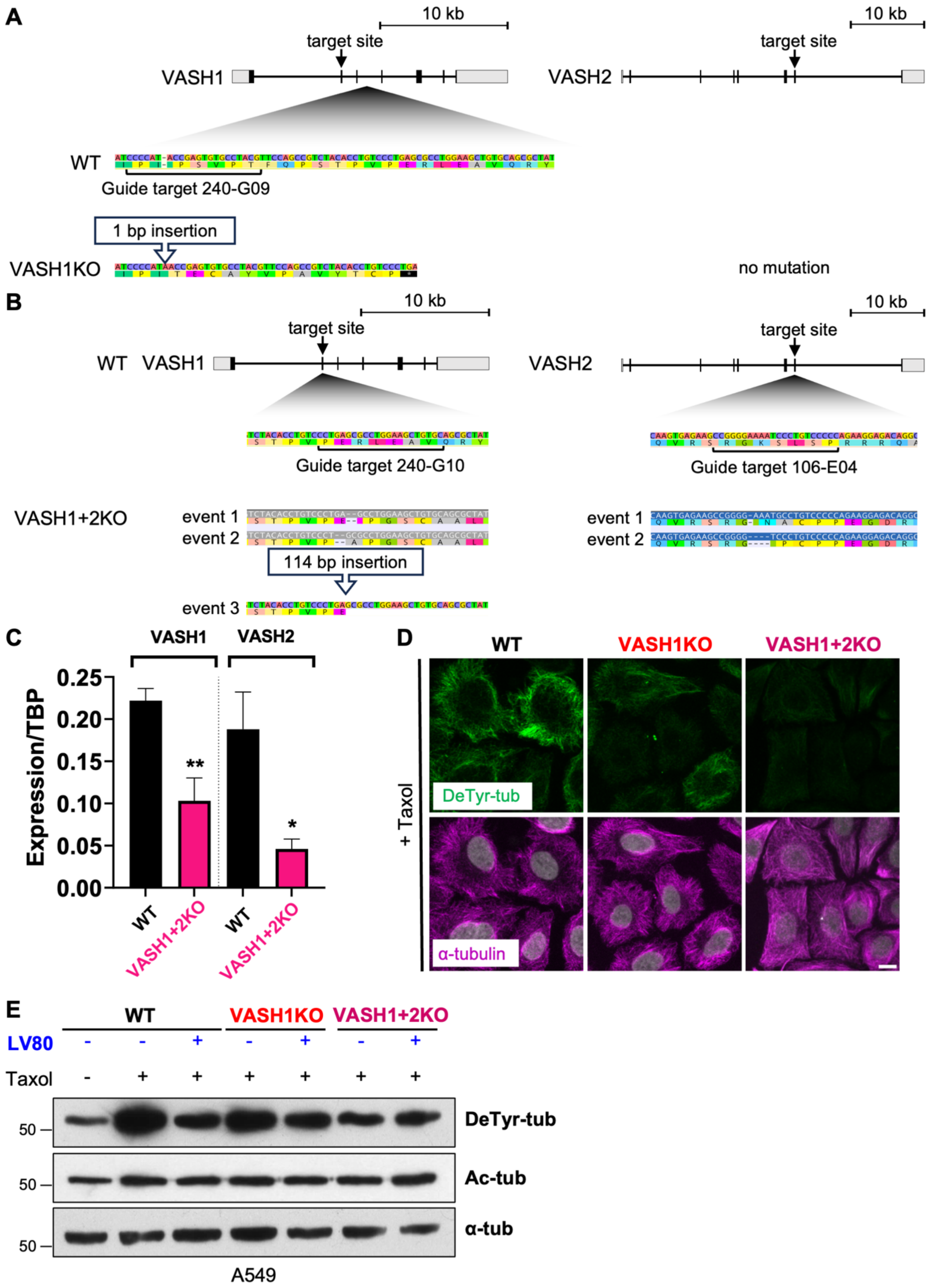

A
10 kb
target site
VASH1
VASH2
WT
Guide target 240-G09
1 bp insertion
VASH1KO
no mutation
B
WT VASH1
VASH2
Guide target 240-G10
Guide target 106-E04
VASH1+2KO
event 1
event 2
114 bp insertion
event 3
C
VASH1
VASH2
Expression/TBP
0.25
0.20
0.15
0.10
0.05
0.00
**
*
WT
VASH1+2KO
D
WT
VASH1KO
VASH1+2KO
+ Taxol
DeTyr-tub
α-tubulin
E
LV80
Taxol
50
DeTyr-tub
Ac-tub
α-tub
A549

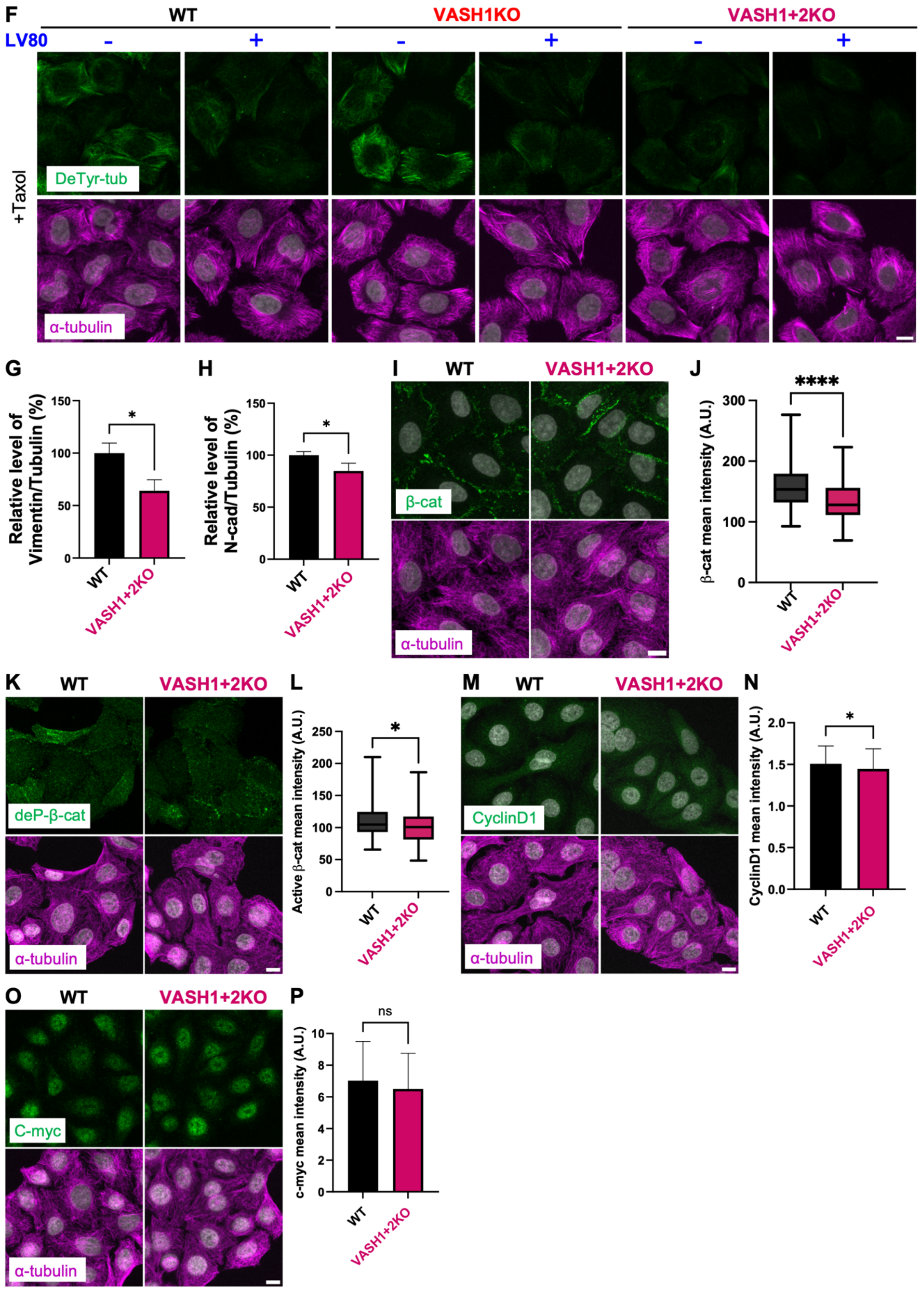

Generation and phenotypical characterization of A549 VASH knockout cell lines (related to Figure 4). (**A**) Drawing representing the structure of the VASH1 and VASH2 loci. Exons are

represented as rectangles and the coding regions are in black. The sequence targeted by the guide for VASH1 is indicated. Nucleotidic and proteinic sequence of the WT and mutated VASH1 alleles are represented below. (**B**) Drawing representing the structure of the VASH1 and VASH2 loci. Exons are represented as rectangles and the coding regions are in black. The sequences targeted by the guides for VASH1 and VASH2 are indicated. Nucleotidic and proteinic sequence of the WT and mutated VASH1 and VASH2 alleles are represented below. (**C**) Graphical representation of ddPCR-based measurements of VASH1 and VASH2 mRNA levels compared to TBP mRNA as a reference in WT or VASH1+2KO A549 cells. (**D**) Immunofluorescence analysis of WT, VASH1-KO and VASH1+2KO A549 cells treated with Taxol co-labelled for detyrosination (DeTyr-tub) and α-tubulin. Note reduced levels of detyrosination in VASH1-KO and complete absence of this modification in VASH1+2KO cells. (**E**) Immunoblot analysis of WT, VASH1-KO and VASH1+2KO A549 cells either treated or not with LV80 in the presence of Taxol. (**F**) Immunofluorescence analysis of WT, VASH1-KO and VASH1+2KO cells either treated or not with LV80 in the presence of Taxol. (**G**) Quantification of vimentin protein levels in WT or VASH1+2KO cells. (**H**) Quantification of N-cad protein levels in WT or VASH1+2KO cells. (**I**) Immunofluorescence analysis of WT and VASH1+2KO A549 cells co-labelled for β-catenin (β-cat) and α-tubulin. Note the absence of re-localization to the nucleus indicative of the lack of activation. (**J**) Quantification of β-catenin fluorescence intensity in WT and VASH1+2KO cells. (**K**) Immunofluorescence analysis of WT and VASH1+2KO A549 cells co-labelled for dephosphorylated β-catenin (deP-β-cat) and α-tubulin. Note the absence of re-localization to the nucleus indicative of the lack of activation. (**L**) Quantification of active (dephosphorylated) beta-catenin fluorescence intensity in WT and VASH1+2KO cells. (**M**) Immunofluorescence analysis of WT and VASH1+2KO A549 cells co-labelled for cyclin D1 and α-tubulin. (**N**) Quantification of cyclinD1 fluorescence intensity in WT or VASH1+2KO cells. (**O**) Immunofluorescence analysis of WT and VASH1+2KO A549 cells co-labelled for C-myc and α-tubulin. (**P**) Quantification of c-myc fluorescence intensity in WT or VASH1+2KO cells. Scale bars in all panels are 10 μm.

Fig. S5.

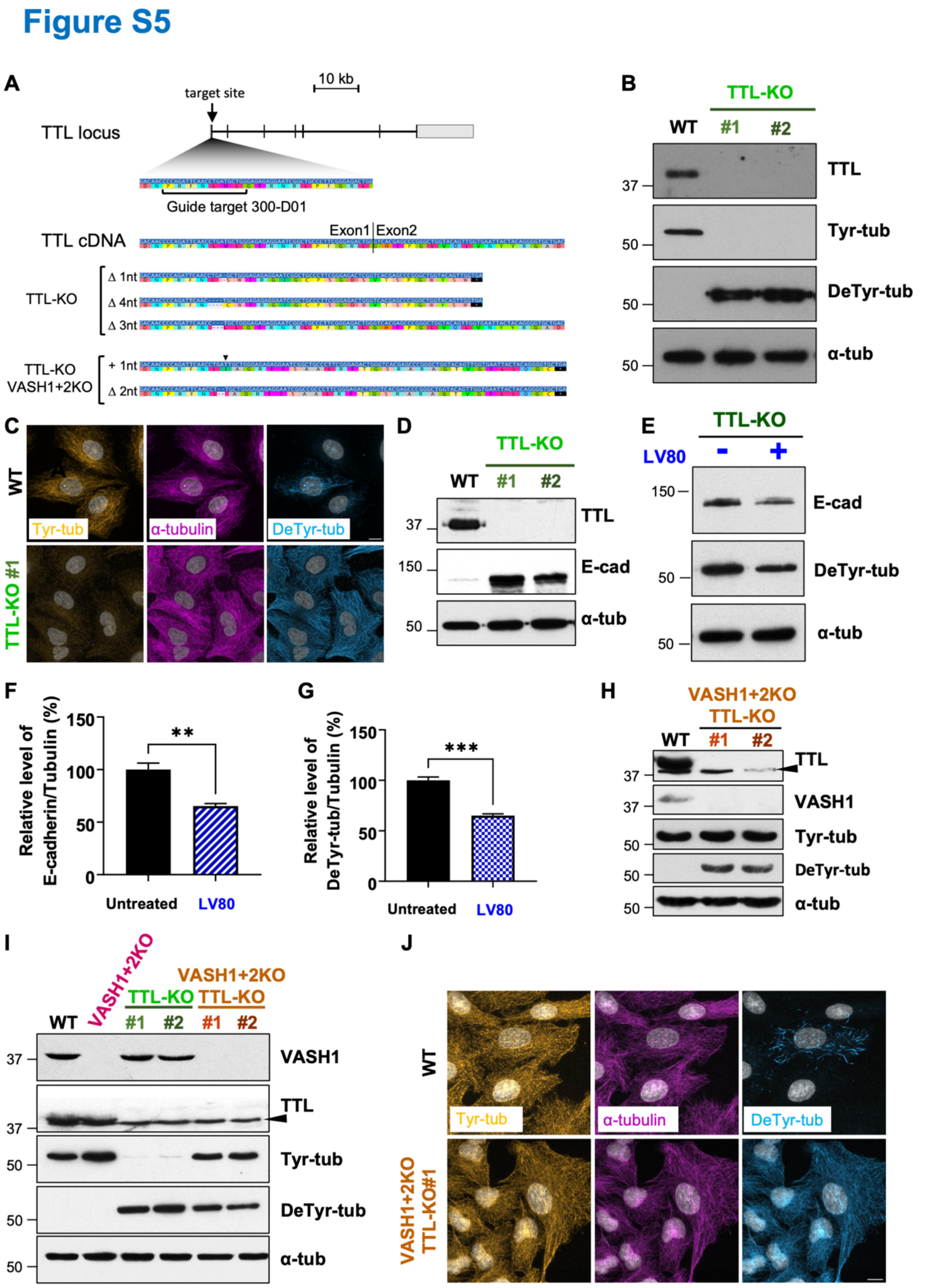

Figure S5
A
target site
10 kb
TTL locus
Guide target 300-D01
TTL cDNA
Exon1
Exon2
TTL-KO
Δ 1nt
Δ 4nt
Δ 3nt
TTL-KO VASH1+2KO
+ 1nt
Δ 2nt
B
TTL-KO
WT
#1
#2
TTL
Tyr-tub
DeTyr-tub
α-tub
37
50
C
WT
TTL-KO #1
Tyr-tub
α-tubulin
DeTyr-tub
D
TTL-KO
WT
#1
#2
TTL
E-cad
α-tub
37
150
50
E
TTL-KO
LV80
-
+
E-cad
DeTyr-tub
α-tub
150
50
F
Relative level of E-cadherin/Tubulin (%)
150
100
50
0
**
Untreated
LV80
G
Relative level of DeTyr-tub/Tubulin (%)
150
100
50
0
***
Untreated
LV80
H
VASH1+2KO
TTL-KO
WT
#1
#2
TTL
VASH1
Tyr-tub
DeTyr-tub
α-tub
37
50
I
WT
VASH1+2KO
TTL-KO
VASH1+2KO TTL-KO
#1
#2
VASH1
TTL
Tyr-tub
DeTyr-tub
α-tub
37
50
J
WT
VASH1+2KO TTL-KO#1
Tyr-tub
α-tubulin
DeTyr-tub

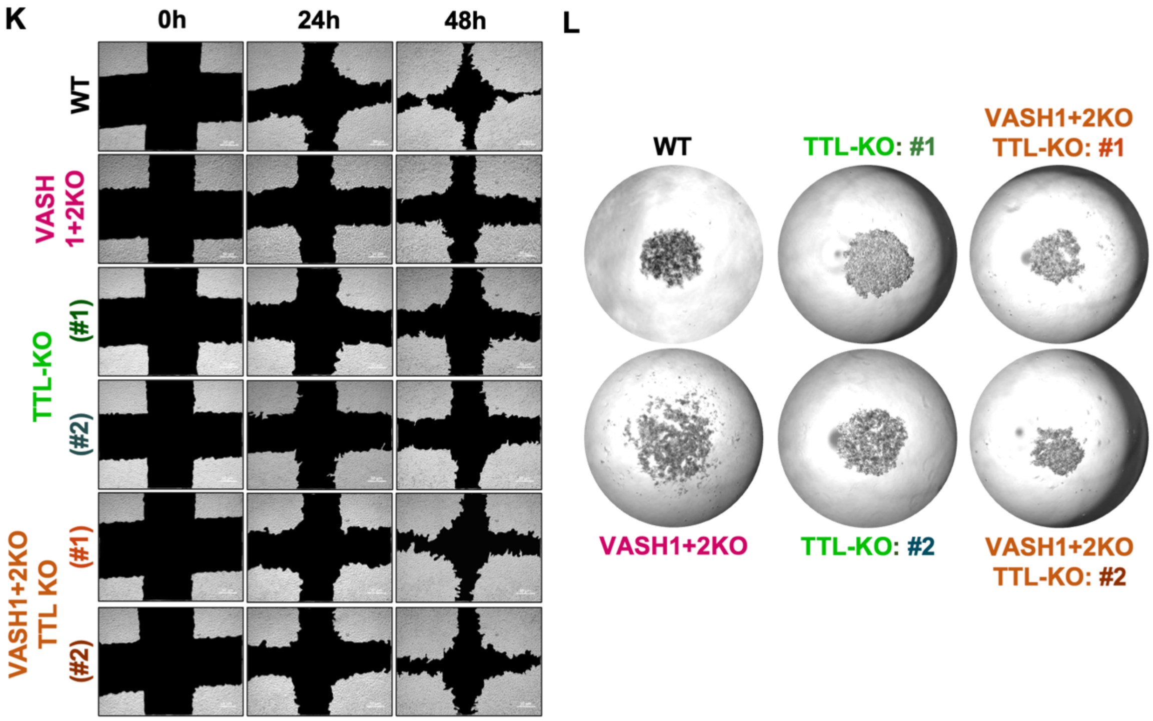

Rescue of the VASH1+2KO phenotypes by TTL knockout in A549 cells (related to Figure 5). (**A**) Drawing representing the structure of the TTL locus. Exons are shown as rectangles and the coding regions are in black. The sequence targeted by the guide for TTL is indicated. Nucleotidic and proteinic sequence of the WT and mutated TTL alleles in TTL-KO and VASH-2KO TTL-KO cells are represented below. (**B**) Immunoblot analysis of protein extracts from either WT or two independent clones (#1 and #2) knockout for TTL (TTL-KO). Note the complete absence of tyrosinated tubulin in the TTL-KO cells. (**C**) Immunofluorescence analysis of WT and TTL-KO A549 cells co-labelled for tyrosinated tubulin (Tyr-tub), α-tubulin and detyrosination (DeTyr-tub). Note the complete absence of the labelling for tyrosinated tubulin in the TTL-KO cells. (**D**) Graphical representation of ddPCR-based measurements of E-cad mRNA levels compared to TBP mRNA as a reference in WT and TTL-KO A549 cells. (**E)** Immunoblot analysis of protein extracts from TTL-KO cells treated or not with LV80 for 72h. Note the reduction in E-cad protein levels upon LV80 treatment. (**F**) Quantification of E-cad protein levels in A549 TTL-KO cells treated with LV80. (**G**) Quantification of detyrosination levels in A549 TTL-KO cells treated with LV80. (**H**) Immunoblot analysis of WT and VASH1+2KO TTL-KO cells. The arrowhead points at an unspecific band recognized by the anti-TTL antibodies. (**I**) Immunoblot analysis of protein extracts from WT, VASH1+2KO, TTL-KO and VASH1+2KO TTL-KO A549 cells. Note that while tyrosinated tubulin is completely absent from TTL-KO clones, its presence is not affected in VASH1+2KO TTL-KO clones. (**J**) Immunofluorescence analysis of WT or VASH1+2KO TTL-KO A549 cells co-labelled for tyrosinated tubulin, α-tubulin and detyrosination. Note the even distribution of detyrosination on all MTs in VASH1+2KO TTL-KO cells. (**K**) Comparison of the migration speed of WT, VASH1+2KO, TTL-KO and VASH1+2KO TTL-KO cells using wound healing assay. (**L**) Comparison of the ability of WT, VASH1+2KO, TTL-KO and VASH1+2KO TTL-KO cells to form a compact spheroid after 48h using hanging drop assay. Scale bars in all panels are 10 µm.

Fig. S6.

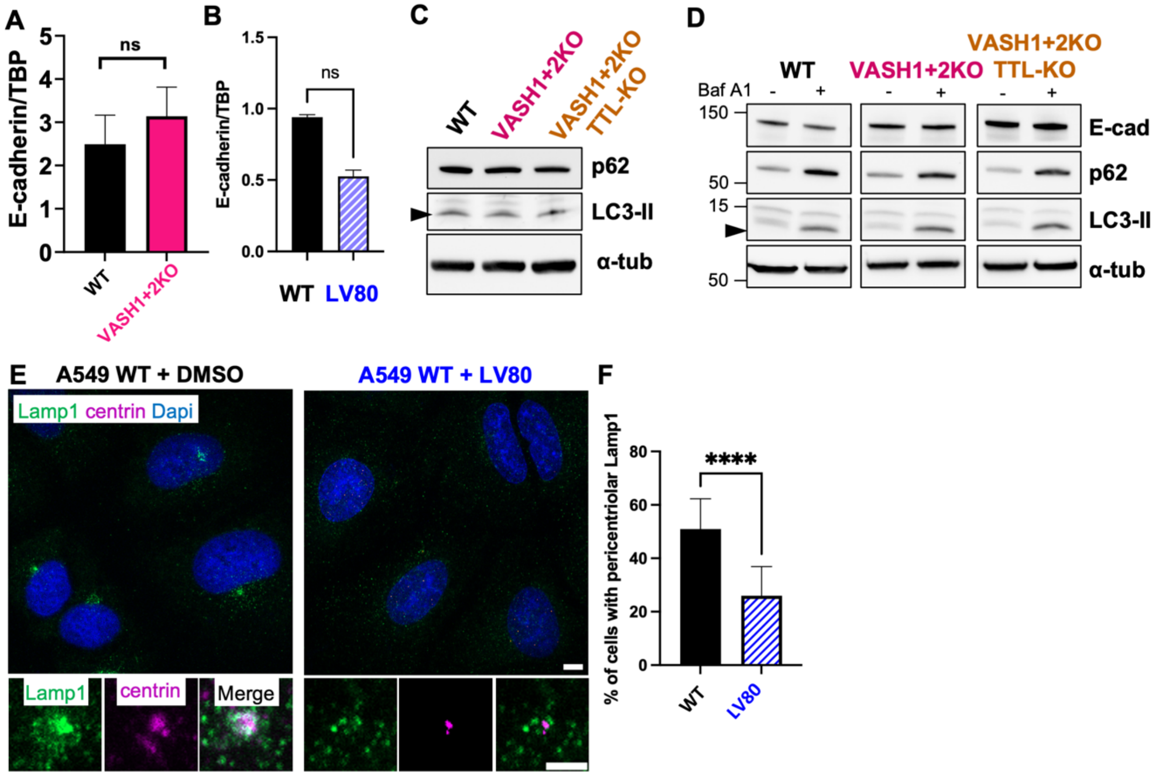

Reduced tubulin detyrosination inhibits lysosome-dependent E-cad degradation (related to Figure 6). (**A**) ddPCR-based quantification of E-cad mRNA levels compared to TBP mRNA as a reference in WT and VASH1+2KO A549 cells. (**B**) ddPCR-based measurements of E-cad mRNA levels compared to TBP mRNA as a reference in A549 cells treated or not with LV80. (**C**) Immunoblot analysis of protein extracts from WT, VASH1+2KO or VASH1+2KO TTLKO A549 cells. (**D**) Immunoblot analysis of protein extracts from WT, VASH1+2KO or VASH1+2KO TTLKO A549 cells treated of not with Bafilomycin A1 (BafA1) for 4h. Note the similar increase in LC3-II (arrowhead) and p62 levels upon BafA1 treatment in all three genotypes. (**E**) Immunofluorescence analysis of WT A549 cells treated or not with LV80 for 48h, co-labelled for Lamp1 and centrin. Note the loss of pericentriolar accumulation of Lamp1 in LV80-treated cells. (**F**) Percentage of cells exhibiting Lamp1 pericentriolar accumulation.

Fig. S7.

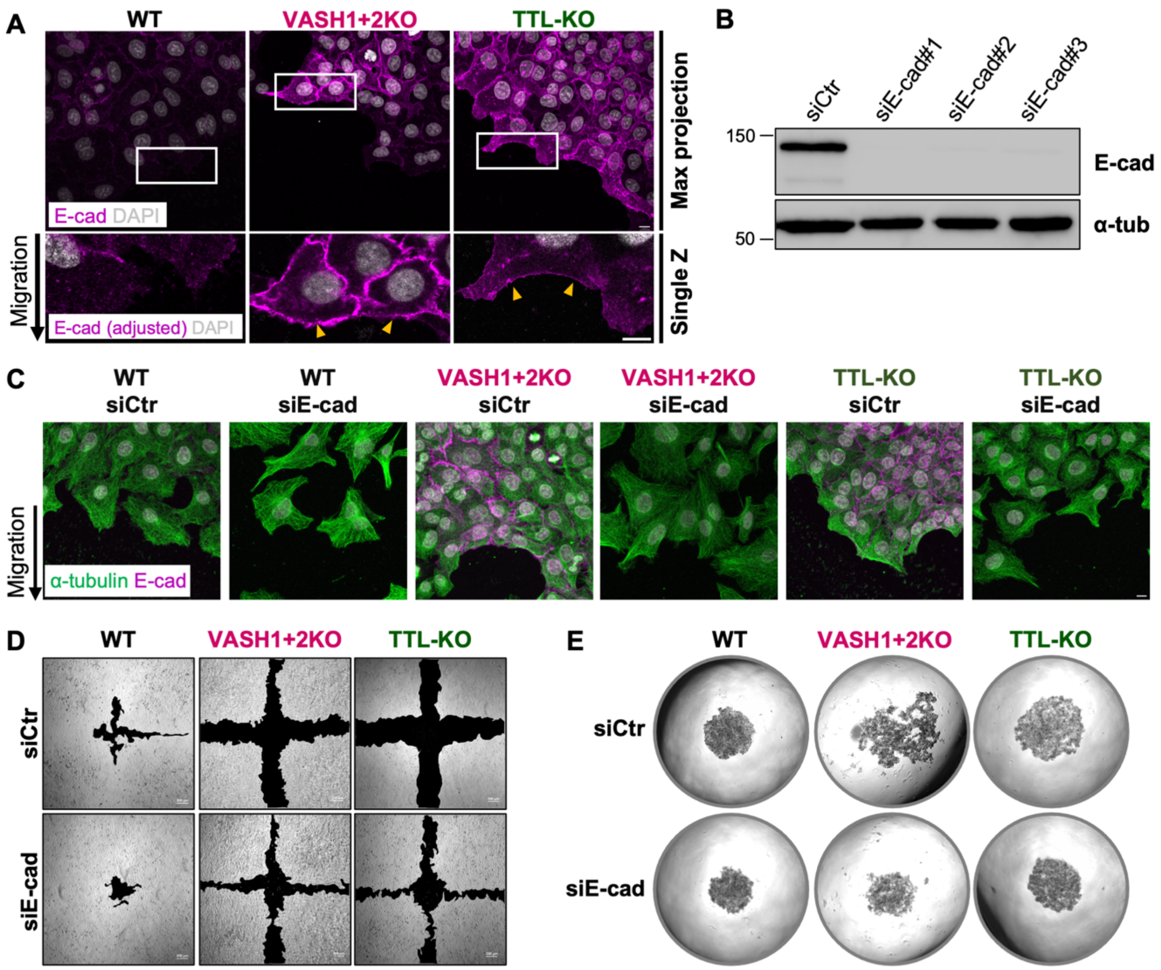

Evaluation of the role of E-cadherin in cell migration and spheroid formation (related to Figure 7). (**A**) Immunofluorescence analysis of the migrating front of WT, VASH1+2KO and TTL-KO A549 cells labelled for E-cad after wound healing assay. The insets (bottom) represent a single Z plane showing the presence of E-cad at the migrating front in VASH1+2KO and TTL-KO but not WT cells. Scale bar: 10 µm. (**B**) Immunoblot analysis of protein extracts from A549 cells transfected with either control siRNA (siCtr) or three different siRNAs against E-cad (siE-cad#1-3). Transfection with all three siRNAs against E-cad leads to efficient depletion of the targeted protein. (**C**) Immunofluorescence analysis of WT, VASH1+2KO and TTL-KO A549 cells transfected with either control or E-cad siRNA co-labelled for E-cad and α-tubulin. The efficiency of the E-cad knockdown is underscored by a complete disappearance of the E-cad labelling in VASH1+2KO and TTL-KO cell lines. Scale bar is 10 µm. (**D**) Comparison of the migration speed of the WT, VASH1+2KO and TTL-KO A549 cells after 48h using wound healing assay. Scale bar: 200 µm. (**E**) Comparison of the ability of WT, VASH1+2KO and TTL-KO A549 cells transfected either with siCtr or siE-Cad to form a compact spheroid after 48h using hanging drop assay.

Tables

Table S1. Sequences of gRNAs used for the generation of knockout cell lines.

Gene	Clone number	HCRISPR#	Guide sequence
VASH1	VASH1-KO<br><br>VASH1+2KO	240-G09<br><br>240-G10	CGTAGGCACACTCGGTATGGGG (Exon 4)<br>GCACAGCTTCCAGGCGCTCAGG (Exon 4)
VASH2	VASH1+2KO	106-E04	GGGGGACAGGGATTTTCCCCG G (Exon 7)
TTL	TTL-KO #1 and #2<br>VASH1+2KO/TTL-KO #1 and #2	300-D01	CCCAGATTCAACCTGATGCTGG (Exon 1)

Table S2. Primers used for the amplification of DNA prior to sequencing.

Gene	HCRISPR #	Primer Forward	Primer Reverse
VASH1 (Genomic)	240-G09<br>240-G10	AGCCACAGAGCTACAGCAAG<br>Same primer	CCTGTCAGAGGTCTGCTCTTC<br>Same primer
VASH2 (Genomic)	106-E04	GCTGGTCCTCAACGTCTCAA	TAGCCACCATTCTGTGCAGG
TTL (cDNA)	300-D01	CTTCTCGGCCGCCTGGTC	AATGAGCCACACCTTCAGCT

Table S3. Statistical tests and p-values.

Figure panel	n	Statistical test	p-value	Significance
Fig. 1G – IC50 EpoY vs. LV80	3	One-way Anova test	**EpoY**<br>$p>0.9999$<br>$p=0.9989$<br>$p=0.7289$<br>$p=0.6653$<br>$p=0.0646$<br>$p<0.01$<br>$p<0.0001$<br><br>**LV80**<br>$p>0.9999$<br>$p=0.7946$<br>$p<0.05$<br>$p<0.0001$	 ns (0 vs. 0.01)<br>ns (0 vs. 0.05)<br>ns (0 vs. 0.1)<br>ns (0 vs. 0.5)<br>ns (0 vs. 1)<br>**<br>****<br><br><br>ns (0 vs. 0.01)<br>ns (0 vs. 0.05)<br>*<br>****
Fig. 2C – IC50 biotinylated LV80	3	One-way Anova test	$p>0.9999$<br>$p=0.9995$<br>$p= 0.9200$<br>$p= 0.9995$<br>$p= 0.0802$<br>$p<0.0001$	ns (0 vs. 0.01)<br>ns (0 vs. 0.05)<br>ns (0 vs. 0.1)<br>ns (0 vs. 0.5)<br>ns (0 vs. 1)<br>****
Fig. 2G – PTL viability	3	One-way Anova test	$p<0.001$<br>$p<0.0001$	***<br>****
Fig. 2G – Taxol viability	3	One-way Anova test	$p<0.05$<br>$p<0.0001$	*<br>****
Fig. 2G – LV80 viability	3	One-way Anova test	$p=0.5766$<br>$p=0.3282$<br>$p=0.2097$<br>$p=0.2010$<br>$p=0.1284$	ns (0 vs. 0.01)<br>ns (0 vs. 0.1)<br>ns (0 vs. 1)<br>ns (0 vs. 10)<br>ns (0 vs. 100)
Fig. 3B – Detyr levels LV80	9	One-way Anova test	$p<0.0001$	****
Fig. 3C – E-cad levels LV80	5	One-way Anova test	$p<0.05$<br>$p<0.01$	*<br>**
Fig. 3F – Wound healing LV80	4	Two-Way ANOVA test	$p<0.001$<br>$p<0.0001$	***<br>****
Fig. 3H – Spheroid LV80	3	One-way Anova test	$p<0.05$<br>$p<0.001$<br>p<0.0001	*<br>***<br>****
Fig. S3A – LV80 viability A549	3	One-way Anova test	$p=0.9994$<br>$p=0.9996$<br>$p=0.9769$<br>$p=0.9999$	ns (0 vs. 5)<br>ns (0 vs. 25)<br>ns (0 vs. 50)<br>ns (0 vs. 100)
Fig. S3B – LV80 viability IMR-90	3	One-way Anova test	$p=0.9992$<br>$p>0.9999$<br>$p=0.7698$<br>$p=0.9998$	ns (0 vs. 5)<br>ns (0 vs. 25)<br>ns (0 vs. 50)<br>ns (0 vs. 100)
Fig. S3C – N-cad levels LV80	6	One-way Anova test	$p<0.001$	***
Fig. S3D – Vimentin levels LV80	4	One-way Anova test	$p<0.01$	**

Fig. S3F – Detyr levels H1299	3	One-way Anova test	$p<0.01$	**
Fig. S3F – E-cad levels H1299	3	One-way Anova test	$p>0.9999$<br>$p<0.05$<br>$p<0.01$	ns (0 vs. 5)<br>*<br>**
Fig. S3H – Detyr levels H520	3	One-way Anova test	$p=0.3507$<br>$p=0.1387$<br>$p<0.05$	ns (0 vs. 5)<br>ns (0 vs. 25)<br>*
Fig. S3H – E-cad levels H520	4	One-way Anova test	$p=0.3656$<br>$p=0.0756$<br>$p<0.05$<br>$p<0.001$	ns (0 vs. 5)<br>ns (0 vs. 25)<br>*<br>***
Fig. S3J – Detyr levels H1975	3	One-way Anova test	$p=0.2580$<br>$p=0.2736$<br>$p<0.05$	ns (0 vs. 5)<br>ns (0 vs. 25)<br>*
Fig. S3J – E-cad levels H1975	4	One-way Anova test	$p=0.9515$<br>$p=0.3551$<br>$p=0.2060$<br>$p<0.05$	ns (0 vs. 5)<br>ns (0 vs. 25)<br>ns (0 vs. 50)<br>*
Fig. S3L – Wound healing H1299	3	Two-Way ANOVA test	$p<0.01$<br>$p<0.001$	**<br>***
Fig. S3N – Spheroid H1299	3	t-test	$p<0.01$	**
Fig. S3P – Detyr levels drops	3	One-way Anova test	$p=0.9995$<br><br>$p=0.0700$<br><br>$p<0.05$<br>$p<0.01$	ns (Mono vs. Drop 24h)<br>ns (Drop 24h vs. Drop 48h)<br>*, #<br>**
Fig. 4D – Wound healing V1+2KO	3	Two-Way ANOVA test	$p= 0.1981$<br><br><br><br>$p= 0.5636$<br><br><br><br><br>$p<0.01$<br>$p<0.001$	ns (WT LV80 vs. VASH1+2KO untreated)<br>ns (VASH1+2KO untreated vs. VASH1+2KO LV80)<br>**<br>###
Fig. 4F – Spheroid V1+2KO	5	Two-Way ANOVA test	$p= 0.3558$<br><br><br><br>$p=0.2547$<br><br><br><br>$p<0.0001$	ns (WT-LV80 vs. VASH1+2KO untreated)<br>ns (VASH1+2KO untreated vs. LV80)<br>****, ####
Fig. 4H – Blot E-cad V1+2KO	3	t-test	$p<0.05$	*
Fig. S4C – VASH1 mRNA V1+2KO	3	t-test	$p<0.01$	**
Fig. S4C – VASH2 mRNA V1+2KO	3	t-test	$p<0.05$	*
Fig. S4G – Blot Vimentin V1+2KO	3		$p<0.05$	*
Fig. S4H – Blot N-cad V1+2KO	3		$p<0.05$	*
Fig. S4J – beta-cat V1+2KO	2	t-test	$p<0.0001$	****

	WT: 111 cells<br>2KO: 140 cells			
Fig. S4L – active beta-cat V1+2KO	2<br>WT: 111 cells<br>2KO: 140 cells	t-test	$p<0.05$	*
Fig. S4N – cyclinD1 V1+2KO	2<br>WT: 141 cells<br>2KO: 96 cells	t-test	$p<0.05$	*
Fig. S4P – c-myc V1+2KO	3<br>WT: 243 cells<br>2KO: 171 cells	t-test	$p=0.0530$	ns
Fig. 5C – E-cad levels rescue	3	One-way Anova test	$p=0.8965$<br>$p<0.01$	ns<br>**, ##
Fig. 5E – Wound healing rescue	8	One-way Anova test	$p<0.01$<br>$p<0.001$<br>$p<0.0001$	**, ##<br>***, ###<br>****
Fig. 5G – Spheroid rescue	5	One-way Anova test	$p=0.9523$<br><br><br>$p>0.9999$<br><br><br>$p<0.0001$	ns (WT vs. VASH1+2 TTL-KO #1<br>ns (WT vs. VASH1+2 TTL-KO #2<br>****, ####
Fig. S5F – E-cad levels TTLKO+LV80	3	t-test	$p<0.01$	**
Fig. S5G – detyr levels TTLKO+LV80	3	t-test	$p<0.001$	***
Fig. 6A – E-cad mRNA V1+2KO	3	t-test	$p>0.999$	ns
Fig. 6C – CHX (T48h only)	2	Paired t-test	$p=0,0412$	*
Fig. 6E – Lamp1 perinuclear localization	3<br>WT: 381cells<br>2KO: 525 cells<br>Rescue: 604 cells	One-way Anova test	$p=0,4494$<br><br><br>$p<0.0001$	ns (WT vs. VASH1+2 TTL-KO)<br>****
Fig. 6G – Lamp1 perinuclear localization LV80	2<br>WT: 209 cells<br>LV80: 191 cells<br>Rescue: 604 cells	t-test	$p<0.0001$	****
Fig. S6A – E-cad mRNA LV80	3	Mann-Whitney test	$p=0.1$	ns
Fig. 7D – Wound healing siE-cad	5	Two-Way ANOVA test	$p=0.995$<br>$p<0.0001$	ns<br>****
Fig. 7F – Spheroid siE-cad	3	Two-Way ANOVA test	$p=0.997$<br>$p<0.0001$	ns<br>****

SI References

1. J. Wehland, M. C. Willingham, A rat monoclonal antibody reacting specifically with the tyrosylated form of alpha-tubulin. II. Effects on cell movement, organization of microtubules, and intermediate filaments, and arrangement of Golgi elements. *J Cell Biology* **97**, 1476–1490 (1983).

2. J. Schindelin, *et al.*, Fiji: an open-source platform for biological-image analysis. *Nat Methods* **9**, 676–682 (2012).

3. G. Gillard, G. Girdler, K. Röper, A release-and-capture mechanism generates an essential non-centrosomal microtubule array during tube budding. *Nat Commun* **12**, 4096 (2021).

4. K. Ersfeld, *et al.*, Characterization of the tubulin-tyrosine ligase. *J Cell Biology* **120**, 725–732 (1993).

5. K. Rogowski, *et al.*, A Family of Protein-Deglutamylating Enzymes Associated with Neurodegeneration. *Cell* **143**, 564–578 (2010).

6. S. Nicot, *et al.*, A family of carboxypeptidases catalyzing α- and β-tubulin tail processing and deglutamylation. *Sci. Adv.* **9**, eadi7838 (2023).

7. R. M. Corrales, *et al.*, Tubulin detyrosination shapes Leishmania cytoskeletal architecture and virulence. *Proc. Natl. Acad. Sci.* **122** (2025).

8. K. Rogowski, *et al.*, A Family of Protein-Deglutamylating Enzymes Associated with Neurodegeneration. *Cell* **143**, 564–578 (2010).

9. G. M. Morris, *et al.*, AutoDock4 and AutoDockTools4: Automated docking with selective receptor flexibility. *J. Comput. Chem.* **30**, 2785–2791 (2009).

10. S. Liao, *et al.*, Molecular basis of vasohibins-mediated detyrosination and its impact on spindle function and mitosis. *Cell Res* **29**, 533–547 (2019).

11. M. H. M. Olsson, C. R. Søndergaard, M. Rostkowski, J. H. Jensen, PROPKA3: Consistent Treatment of Internal and Surface Residues in Empirical pK a Predictions. *J. Chem. Theory Comput.* **7**, 525–537 (2011).

12. S. Kim, *et al.*, CHARMM-GUI ligand reader and modeler for CHARMM force field generation of small molecules. *J. Comput. Chem.* **38**, 1879–1886 (2017).

13. K. Vanommeslaeghe, A. D. MacKerell, Automation of the CHARMM General Force Field (CGenFF) I: Bond Perception and Atom Typing. *J. Chem. Inf. Model.* **52**, 3144–3154 (2012).

14. K. Vanommeslaeghe, E. P. Raman, A. D. MacKerell, Automation of the CHARMM General Force Field (CGenFF) II: Assignment of Bonded Parameters and Partial Atomic Charges. *J. Chem. Inf. Model.* **52**, 3155–3168 (2012).

15. J. Huang, *et al.*, CHARMM36m: an improved force field for folded and intrinsically disordered proteins. *Nat. Methods* **14**, 71–73 (2017).

16. J. Lee, *et al.*, CHARMM-GUI Input Generator for NAMD, GROMACS, AMBER, OpenMM, and CHARMM/OpenMM Simulations Using the CHARMM36 Additive Force Field. *J. Chem. Theory Comput.* **12**, 405–413 (2016).

17. S. Jo, T. Kim, V. G. Iyer, W. Im, CHARMM-GUI: A web-based graphical user interface for CHARMM. *J. Comput. Chem.* **29**, 1859–1865 (2008).

18. D. V. D. Spoel, *et al.*, GROMACS: Fast, flexible, and free. *J. Comput. Chem.* **26**, 1701–1718 (2005).

19. B. Hess, P-LINCS: A Parallel Linear Constraint Solver for Molecular Simulation. *J. Chem. Theory Comput.* **4**, 116–122 (2008).
